# Supplementary material for: Identifying CNS-colonizing T cells as potential therapeutic targets to prevent progression of multiple sclerosis
Source: Med. 2021 Mar 12;2(3):296–312.e8. doi: 10.1016/j.medj.2021.01.006 (PMC7966680; doi:10.1016/j.medj.2021.01.006)
Supplement: Document S1. Figures S1–S7 [file mmc1.pdf]

Med, Volume *2*

Supplemental information

Identifying CNS-colonizing T cells as potential therapeutic targets to prevent progression of multiple sclerosis

Max Kaufmann, Hayley Evans, Anna-Lena Schaupp, Jan Broder Engler, Gurman Kaur, Anne Willing, Nina Kursawe, Charlotte Schubert, Kathrine E. Attfield, Lars Fugger, and Manuel A. Friese

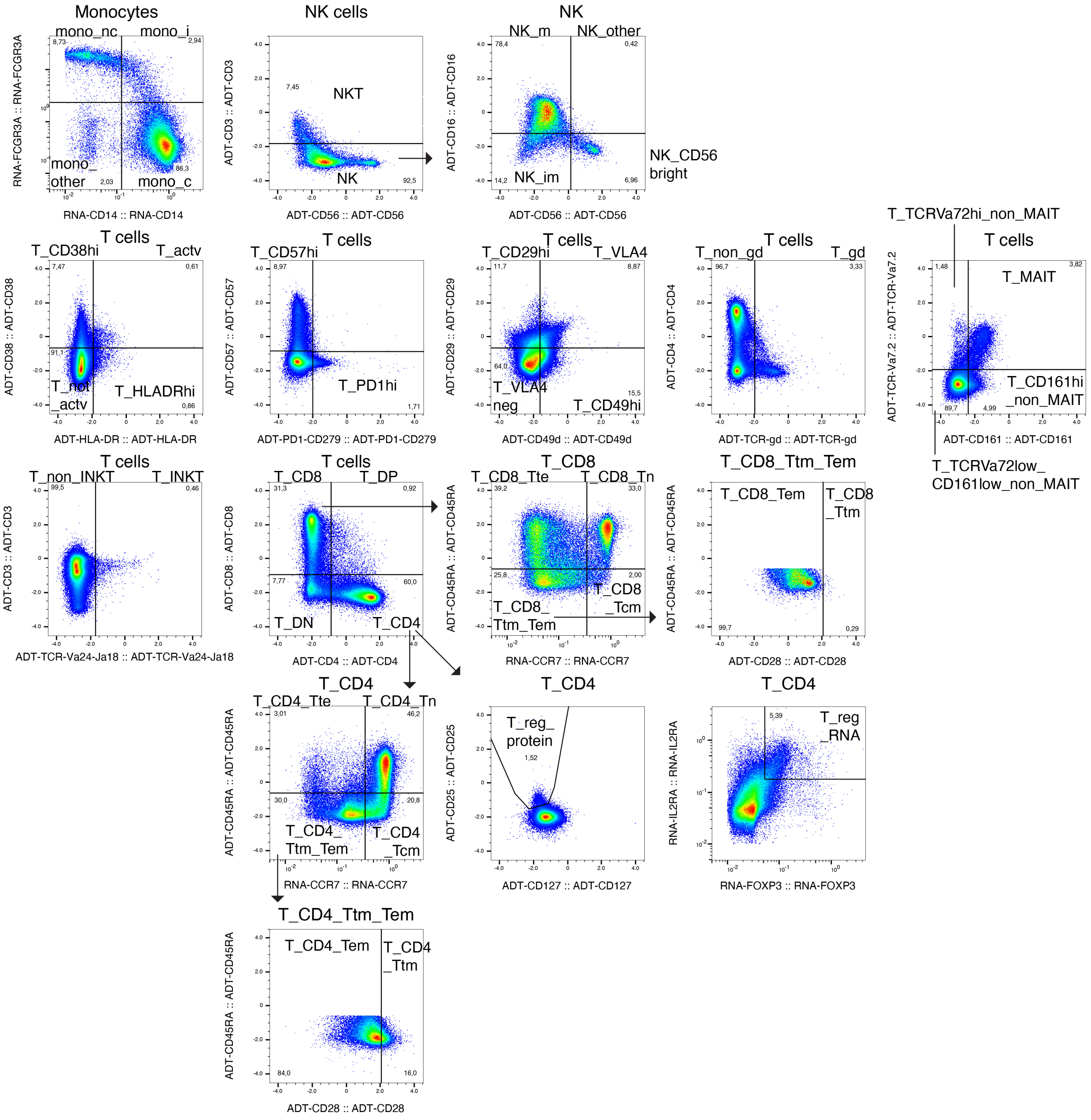

Figure S1. Gating strategy, related to Figure 2

Gating strategy based on sequenced surface marker profiles and imputed mRNA markers. Antibody-derived tags (ADT) indicate that protein surface markers are shown, whereas 'RNA' indicates imputed expression values based on the transcriptome. Abbreviations used: classical monocytes (mono_c, CD14+ monocytes), non-classical monocytes (mono_nc, CD16+ monocytes), intermediate monocytes (mono_i), NK T cells within NK cell clusters (NK_NKT), mature NK cells (NK_m), immature NK cells (NK_im), CD56-bright NK cells (NK_CD56bright), naïve CD4+ T cells (T_CD4_Tn), CD4+ T cells (T_CD4), CD4+ central memory T cells (T_CD4_Tcm), HLADR-high T cells (T_HLADRhi), invariant NK T cells (T_INKT), CD8+ T effector memory cells (T_CD8_Tem), CD8+ T transitional memory / CD8+ T effector memory cells (T_CD8_Ttm_Tem), mucosal-associated invariant T cells (T_MAIT), CD8+ T transitional memory cells (T_CD8_Ttm), CD8+ T cells (T_CD8), naïve CD8+ T cells (T_CD8_Tn), CD8+ central memory T cells (T_CD8_Tcm), CD4+ effector memory T cells (T_CD4_Tem), T regulatory cells based on RNA expression (T_reg_RNA), PD1-high T cells (T_PD1hi), VLA4+ T cells (T_VLA4), CD29-high T cells (T_CD29hi), T regulatory cells based on protein expression (T_reg_protein), activated T cells (T_actv), CD4+ T transitional memory / CD4+ T effector memory cells (T_CD4_Ttm_Tem), CD4+ T transitional memory cells (T_CD4_Ttm), CD38 -high T cells (T_CD38hi), CD49d-high T cells (T_CD49dhi), CD4–/CD8– = double negative T cells (T_DN), NK T cells within T cell clusters (T_NKT), CD8+ terminal effector T cells (T_CD8_Tte), CD57-high T cells (T_CD57hi), gamma-delta T cells (T_gd), CD161-high / non-MAIT cells (T_CD161hi_non_MAIT), CD4+/CD8+ = double positive T cells (T_DP), CD4+ terminal effector T cells (T_CD4_Tte).

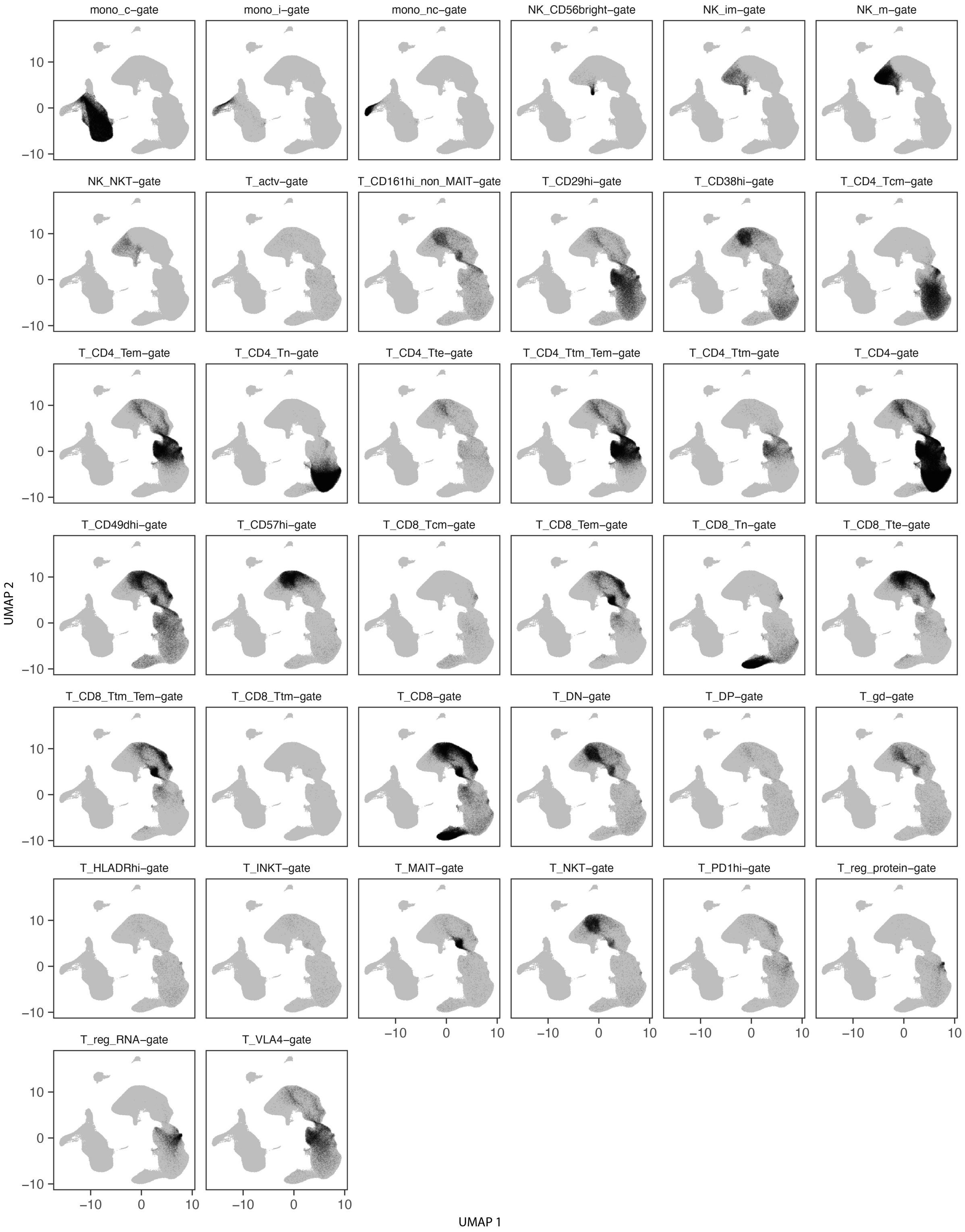

Figure S2. Projection of gates on immune cell map, related to Figure 2

Each gate shown in Figure S1 was projected on umap plots of all cohorts (MS1–3, HI1–3).

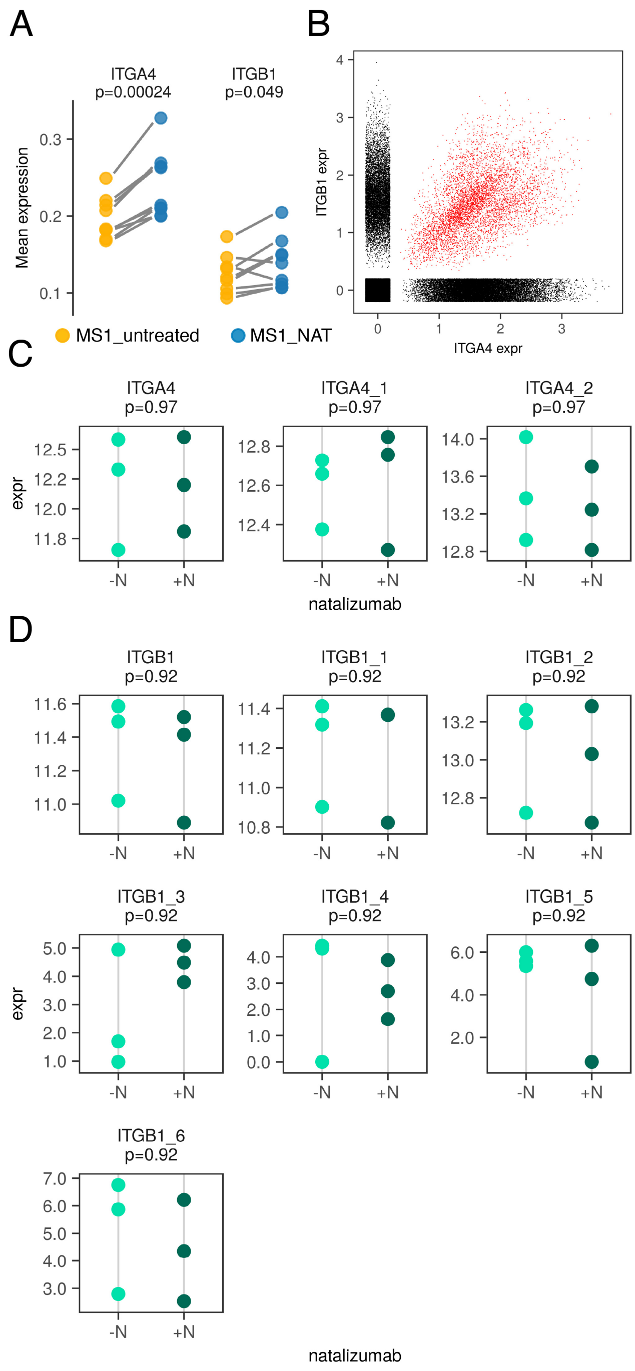

Figure S3. Integrin expression and VLA4 gating, related to Figure 3

(A) Mean expression of ITGA4 and ITGB1 in all PBMCs of n = 9 RRMS patients with and without natalizumab (NAT) treatment (cohort MS1).

(B) Expression of indicated integrins in single PBMCs (cohorts MS1–3, HI1–3). Jitter is added to the data to decrease overplotting. Cells gated as VLA4 positive are marked in red.

(C,D) Microarray-based mRNA expression of indicated integrins from publicly available data[7] in CD4+ T cells from n = 3 healthy donors cultured *in vitro* for 10 days with (+N) or without (–N) natalizumab.

FDR-adjusted paired two-tailed *t*-tests were used in (A). FDR-adjusted unpaired two-tailed *t*-tests were used in (C,D) as data pairs were not indicated in the available metadata.

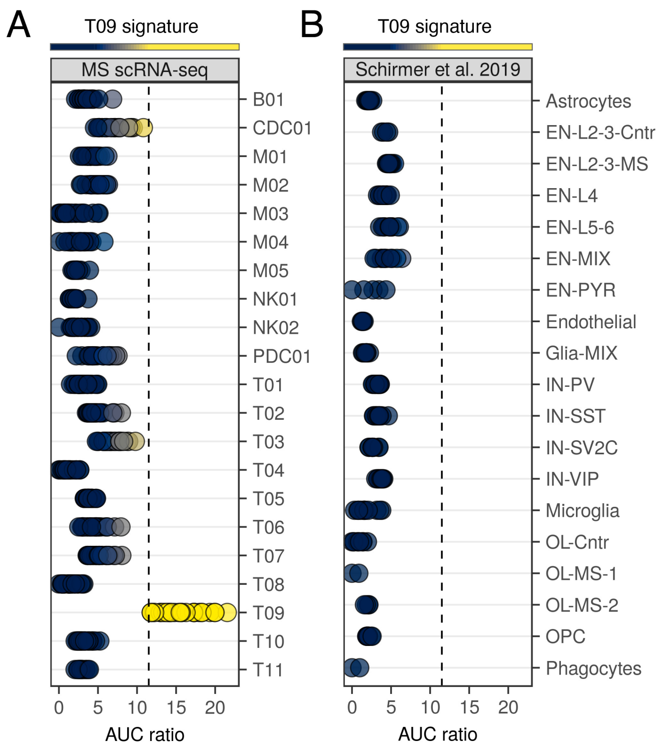

Figure S4. The T09 signature is not enriched in brain cells, related to Figure 6

(A–B) T09 signature enrichment for PBMC scRNA-seq data (A) from *n = 30* MS patients (cohorts MS1–3) and for brain single nuclei RNA sequencing data (B) of *n = 12* MS patients from a publicly available dataset[17].

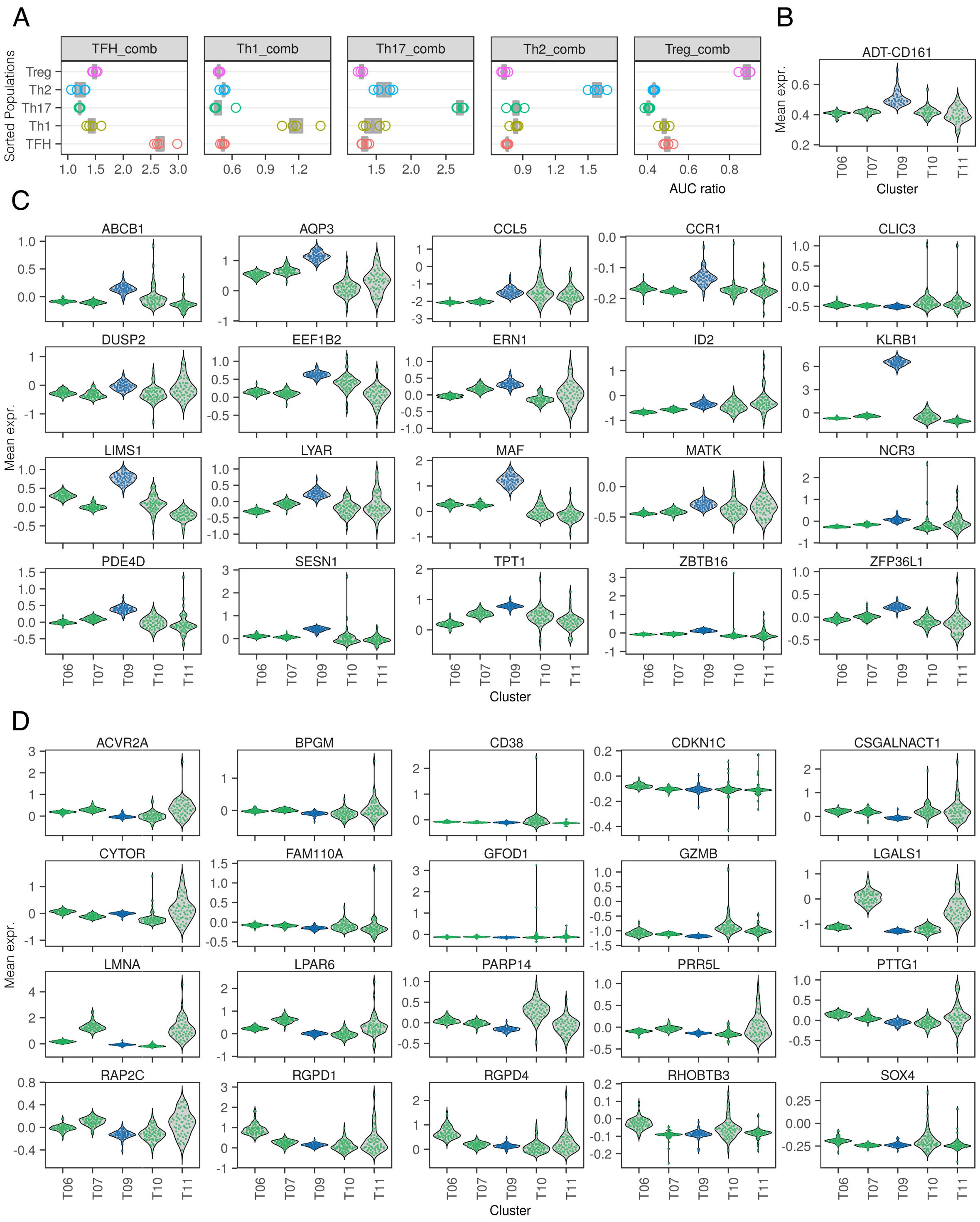

Figure S5. Gene set enrichment-based T helper cell subset discrimination and expression of T09 markers in CD4^{+} memory T cell clusters, related to Figure 7

(A) Marker gene sets for T helper cell subsets were derived from publicly available data[18]. Gene set enrichment of each signature is shown on transcriptomes of sorted T helper cell populations from the same study. Box plots represent median and interquartile range (IQR).

(B) Mean protein surface expression of CD161 per individual (*n* = 42).

(C,D) Mean mRNA expression of indicated genes in CD4mem clusters for *n* = 62 individuals (cohorts MS1–3, HI1–3). (C) Top 20 positive markers for T09, (D) Top 20 negative markers for T09.

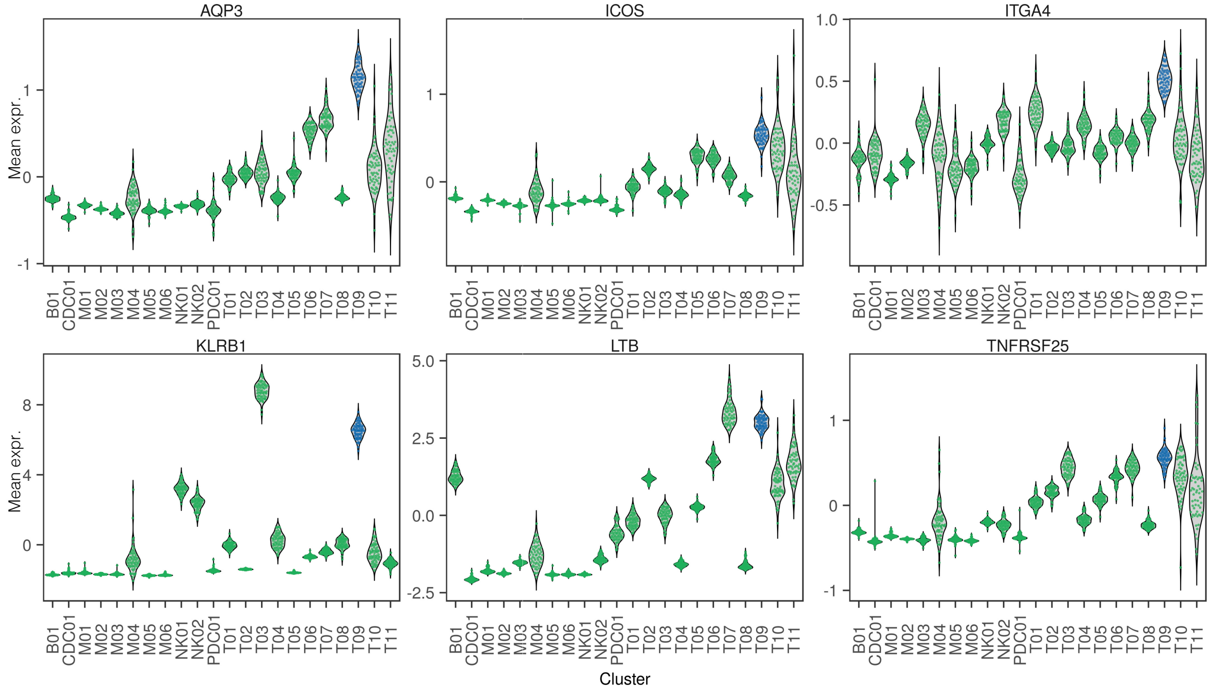

Figure S6. Expression of selected T09 surface markers in all PBMC clusters, related to Figure 7

Mean mRNA expression of indicated genes in indicated clusters for *n* = 62 individuals (cohorts MS1–3, HI1–3).

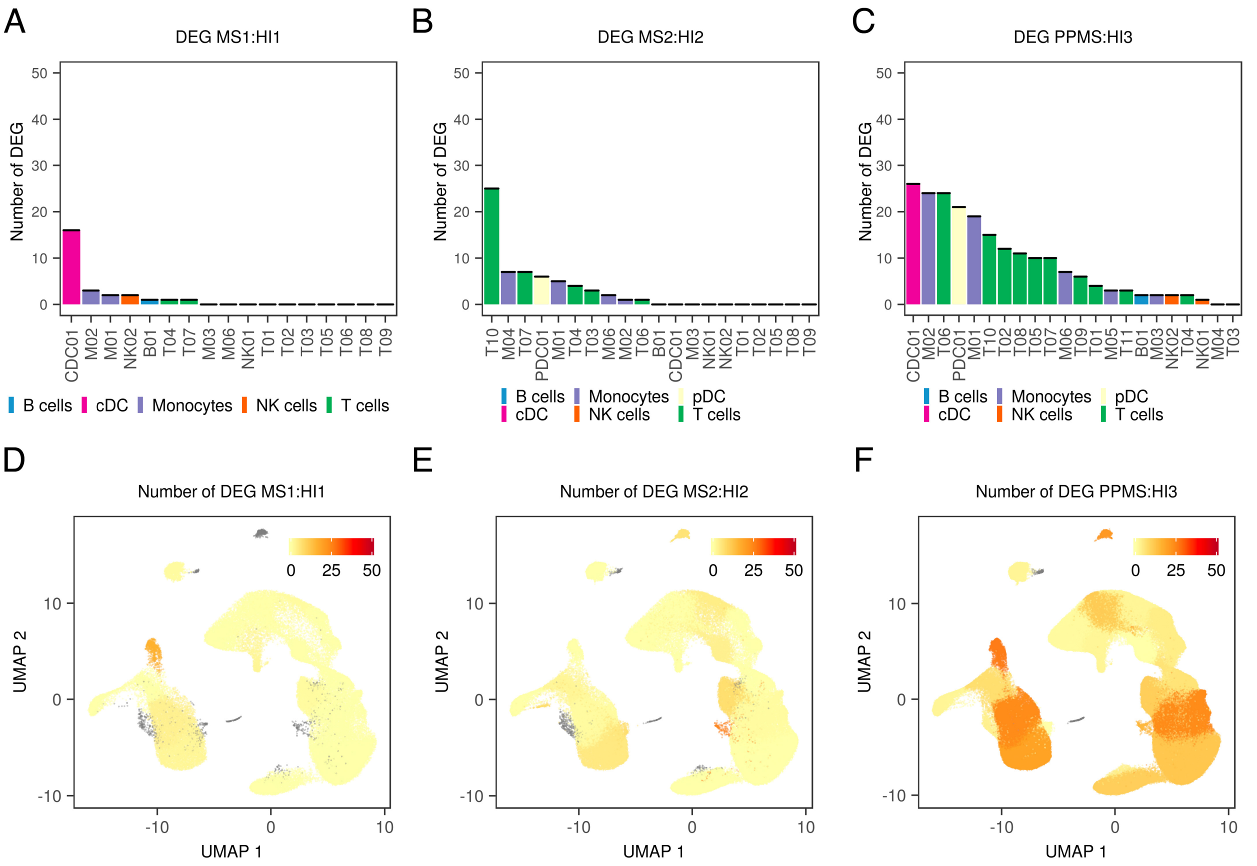

Figure S7. Cross-sectional differential gene expression analysis, related to Figure 4 and limitations of study

(A–C) Number of significantly (adjusted *P* values ≤ 0.1) differentially expressed genes (DEG) between MS patients and healthy individuals. (A) Comparison between cohorts MS1 and HI1, *n* = 19 for T01–04,06–09, NK01,02, M01,02,06, *n* = 18 for T05, M03, *n* = 16 for B01, *n* = 15 for CDC01. (B) Comparison between cohorts MS2 and HI2, *n* = 22 for T01–T09, NK01, M02, *n* = 21 for NK02, *n* = 20 for M01,03,06, *n* = 18 for B01, *n* = 17 for CDC01, *n* = 16 for PDC01, *n* = 15 for M04, *n* = 12 for T10. (C) Comparison between cohorts MS3 and HI3, *n* = 20 for T01–T10, NK01,02, M01–04,06, B01, CDC01, *n* = 19 for M05, PDC01, *n* = 16 for T11.

(D–F) Number of DEG in indicated comparisons projected on umap.
