# Supplementary material for: Identifying CNS-colonizing T cells as potential therapeutic targets to prevent progression of multiple sclerosis
Source: Med. 2021 Mar 12;2(3):296–312.e8. doi: 10.1016/j.medj.2021.01.006 (PMC7966680; doi:10.1016/j.medj.2021.01.006)
Supplement: Document S2. Article plus supplemental information [file mmc6.pdf]

Med

CellPress
OPEN ACCESS

Clinical and Translational Article

Identifying CNS-colonizing T cells as potential therapeutic targets to prevent progression of multiple sclerosis

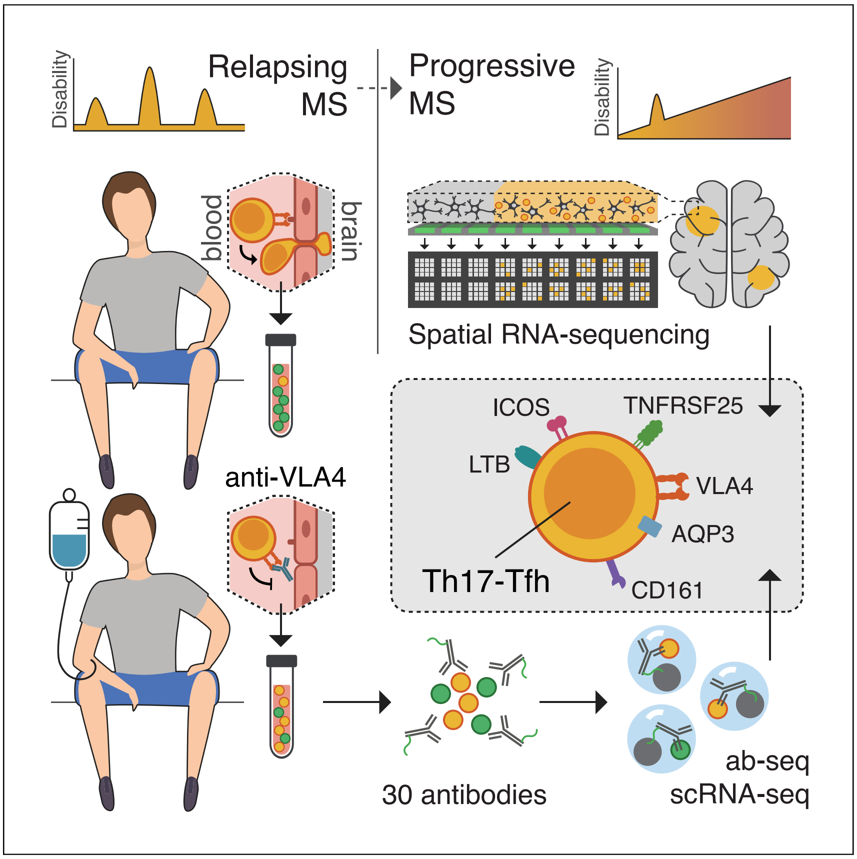

Kaufmann et al. identified pathogenic CD161+/LTB+ T cells resident in brains of MS patients with large-scale multimodal single-cell profiling of blood immune cells and spatial RNA sequencing of brain tissue. They describe targetable surface markers and mobilization to the blood by VLA4-blockage early in MS as prerequisites for therapeutic targeting.

Max Kaufmann, Hayley Evans, Anna-Lena Schaupp, ..., Kathrine E. Attfield, Lars Fugger, Manuel A. Friese

lars.fugger@imm.ox.ac.uk (L.F.)
manuel.friese@zmnh.uni-hamburg.de (M.A.F.)

HIGHLIGHTS

Multimodal single-cell profiling of blood and spatial RNA sequencing of brain tissue

VLA4 blockage mobilizes CD161+/LTB+ Th17/Tfh cells to the blood early in MS

Identification of these Th17/Tfh as brain-resident cells in progressive MS patients

Targetable surface markers on Th17/Tfh include AQP3, TNFRSF25, ICOS, and VLA4

Translation to Patients

Kaufmann et al., Med 2, 296–312
March 12, 2021 © 2021 The Author(s). Published by Elsevier Inc.
https://doi.org/10.1016/j.medj.2021.01.006

CellPress
OPEN ACCESS

Med

Clinical and Translational Article

Identifying CNS-colonizing T cells as potential therapeutic targets to prevent progression of multiple sclerosis

Max Kaufmann,[1,4] Hayley Evans,[2,4] Anna-Lena Schaupp,[2,4] Jan Broder Engler,[1] Gurman Kaur,[2,3] Anne Willing,[1] Nina Kursawe,[1] Charlotte Schubert,[1] Kathrine E. Attfield,[2] Lars Fugger,[2,3,*] and Manuel A. Friese[1,5,*]

SUMMARY

Background: Multiple sclerosis (MS), an autoimmune disease of the central nervous system (CNS), can be suppressed in its early stages but eventually becomes clinically progressive and unresponsive to therapy. Here, we investigate whether the therapeutic resistance of progressive MS can be attributed to chronic immune cell accumulation behind the blood-brain barrier (BBB).

Methods: We systematically track CNS-homing immune cells in the peripheral blood of 31 MS patients and 31 matched healthy individuals in an integrated analysis of 497,705 single-cell transcriptomes and 355,433 surface protein profiles from 71 samples. Through spatial RNA sequencing, we localize these cells in *post mortem* brain tissue of 6 progressive MS patients contrasted against 4 control brains (20 samples, 85,000 spot transcriptomes).

Findings: We identify a specific pathogenic CD161+/lymphotoxin beta (LTB)+ T cell population that resides in brains of progressive MS patients. Intriguingly, our data suggest that the colonization of the CNS by these T cells may begin earlier in the disease course, as they can be mobilized to the blood by usage of the integrin-blocking antibody natalizumab in relapsing-remitting MS patients.

Conclusions: As a consequence, we lay the groundwork for a therapeutic strategy to deplete CNS-homing T cells before they can fuel treatment-resistant progression.

Funding: This study was supported by funding from the University Medical Center Hamburg-Eppendorf, the Stifterverband für die Deutsche Wissenschaft, the OAK Foundation, Medical Research Council UK, and Wellcome.

Context and significance

Multiple sclerosis (MS) is characterized by a rogue immune system that attacks the brain and spinal cord, leading to severe disability in young patients. During early phases of the disease, relapses can still be suppressed by immunomodulatory therapies. Later on, many patients develop a slowly progressive phase that is mostly resistant to available drugs for reasons remaining largely unclear. According to one long-standing theory, the therapeutic resistance might arise due to infiltration and lasting residence of blood immune cells behind the blood-brain barrier, beyond the reach of most therapeutics. In the present study, researchers from the Universities of Hamburg and Oxford joined forces to study this insinuated immune cell colonization of the central nervous system in MS. As a result, they identified a specific immune cell population resident in the brains of progressive MS patients and lay the groundwork to target it therapeutically earlier in the disease course.

INTRODUCTION

MS is a chronic autoimmune disease of the CNS associated with serious physical and cognitive disability. MS manifests most commonly in young adults with a relapsing-remitting disease course (RRMS), in which recurrent episodes (relapses) of neurological symptoms are followed by clinical remission. The majority of MS patients develop a progressive form of the disease, in which an irreversible buildup of disability occurs, regardless of relapses, either from its clinical onset (primary progressive MS [PPMS]) or after approximately 10–20 years of RRMS (secondary progressive MS [SPMS]).[1] Mechanistically, the relapses observed in RRMS have been linked to episodic bouts of CNS invasion by peripheral immune cells that cause

 © 2021 The Author(s). Published by Elsevier Inc.
This is an open access article under the CC BY license (http://creativecommons.org/licenses/by/4.0/).

demyelinating lesions in the white matter.[1] Therefore, targeting of the peripheral immune system can suppress RRMS disease activity as exemplified by the anti-integrin alpha 4 (ITGA4, CD49d) antibody natalizumab.[2] Natalizumab blocks the extravasation of immune cells in the CNS, preventing their migration beyond the BBB and strongly suppressing relapses. Notably, in contrast to RRMS, the same treatment strategies, including natalizumab, are not effective in progressive MS, pointing to a subsequent uncoupling of disease activity from the peripheral immune system.[3] Thus, there is a major unmet clinical need to understand the sequence of events that eventually leads to continuous neurodegeneration and disability progression. The best starting point would be to explore the basis for the uncoupling of the peripheral immune response and disability progression. Although it remains largely unknown and might partly be attributed to primary neurodegeneration, it could be explained by chronic organization of peripheral immune cells behind the BBB, beyond the reach of many common therapeutic strategies. This is suggested by the observation that the onset of progressive MS is best correlated with cortical lesions that are pathologically characterized by lymphocyte collections adjacent to the gray matter.[4–6] However, it is enigmatic whether such colonization of the CNS begins during the RRMS phase and therefore could be prevented at an early stage of disease.

Here, we interrogated the basis of progressive MS by designing a study to (1) systematically identify immune cells homing to the CNS in RRMS, (2) test whether these cells become resident in the CNS of progressive MS patients, and (3) investigate them as a potential therapeutic target. For this purpose, we determined the signature of CNS-homing cells trapped in the blood of natalizumab-treated RRMS patients using a high-dimensional readout of combined single-cell RNA sequencing (scRNA-seq) and protein surface marker profiling. We then tracked these cells in independent cohorts of both untreated RRMS and untreated progressive MS patients and investigated their localization directly in the CNS of progressive patients using spatial RNA sequencing of MS brains. Finally, we describe their detailed characteristics and suggest a strategy for specific targeting with a view to delay or prevent the progressive phase of MS that drives irreversible disability and is untreatable to this day.

RESULTS

An integrated immune cell map of RRMS and progressive MS

To support the discovery of CNS-homing immune cells, we sampled the peripheral blood mononuclear cells (PBMCs) of nine RRMS patients longitudinally with and without natalizumab treatment (cohort MS1; 19 samples total, including 1 additional patient, for which only the treated time point was available). As a basis to study the distribution of these cells in the context of active RRMS and chronic progressive patients, we added two additional untreated cohorts consisting of eleven RRMS patients (cohort MS2) and ten PPMS patients (cohort MS3) as well as age- and sex-matched healthy individuals for all patients in the study (cohorts HI1–3; Figure 1A). All samples were analyzed by multimodal immunophenotyping using scRNA-seq and surface antibody staining, resulting in a comprehensive dataset of 497,705 single-cell transcriptomes and 355,433 surface protein profiles from 71 PBMC samples of 62 donors (Figure 1A). We combined the data of all cells into one integrated immune cell map, which allowed us to establish cell identities across all cohorts and samples (Figures 1B–1F). Unsupervised clustering of the transcriptomic data identified 25 clusters, including 11 *CD3*+ T cell clusters, 6 *FCGR3A* (CD14)+/*CD16*dim monocyte clusters, 1 *CD14*dim/*CD16*+ monocyte cluster, 1 *CD19*+ B cell cluster, 1 *ITM2C*+ plasma cell cluster, 2 *NCAM1* (CD56)+ NK cell clusters, 1 *PPBP*+ megakaryocyte cluster, and 2 *CD14*–, *CD20*–, *HLA-DRA*+ dendritic cell (DC) clusters

[1]Institut für Neuroimmunologie und Multiple Sklerose, Zentrum für Molekulare Neurobiologie Hamburg, Universitätsklinikum Hamburg-Eppendorf, Hamburg, Germany

[2]Oxford Centre for Neuroinflammation, Nuffield Department of Clinical Neurosciences, John Radcliffe Hospital, University of Oxford, Oxford, UK

[3]MRC Human Immunology Unit, Weatherall Institute of Molecular Medicine, John Radcliffe Hospital, University of Oxford, Oxford, UK

[4]These authors contributed equally

[5]Lead contact

*Correspondence:
lars.fugger@imm.ox.ac.uk (L.F.),
manuel.friese@zmnh.uni-hamburg.de (M.A.F.)

https://doi.org/10.1016/j.medj.2021.01.006

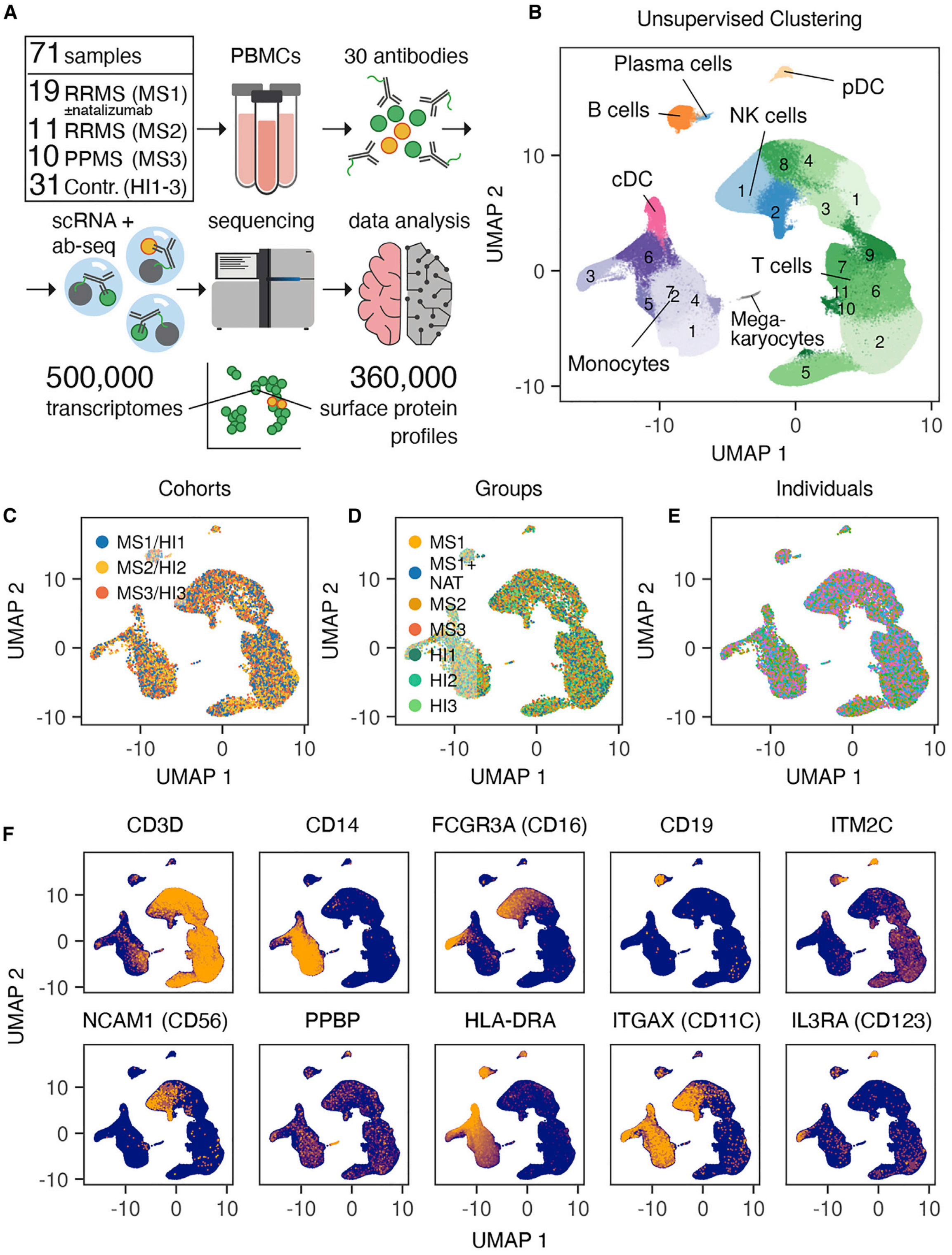

Figure 1. An integrated immune cell map of relapsing-remitting and progressive MS

(A) Schematic overview for multimodal analysis of PBMCs from MS patients (cohorts MS1–3) and healthy individuals (cohorts HI1–3). Cohort MS1 contains samples from time points with and without treatment with natalizumab in 9 patients (for 1 patient, only the treated time point was available).

(B) Unsupervised clustering of n = 497,705 integrated PBMC single cell transcriptomes from n = 62 donors (cohorts MS1–3 and HI1–3).

(C–E) Data integration demonstrated on umap plots.

(consisting of 1 *ITGAX* (CD11c)+ classical DC (cDC) cluster and 1 *IL3RA* (CD123)+ plasmacytoid DC (pDC) cluster; Figures 1B and 1F). Using the surface protein data in combination with imputed RNA-based markers, we defined landmarks of established immune cell populations to augment our unsupervised immune cell map (Figures 2A–2E, S1, and S2). Besides established CD4+ and CD8+ T memory subsets (Figures 2A, 2C, and 2E), we identified specialized T cell subtypes, such as NKT cells, gamma-delta T cells (Tgd), mucosa-associated invariant T cells (MAIT), and T regulatory cells (Treg) (Figures 2A, 2B, 2D, and 2E). Most of the classically defined immune cell populations showed a widespread distribution in the high-dimensional transcriptomic space (Figure 2E), emphasizing the continuous nature of immune cell subtypes.

Defining CNS-homing cells in the peripheral blood of MS patients

In order to identify CNS-homing cells in the blood of RRMS patients, we compared samples from natalizumab-treated time points, where migration into the CNS is hindered, with untreated samples from the same patients (cohort MS1; Figure 3A). We first confirmed that CNS-homing cells were indeed enriched in the peripheral blood by natalizumab by observing increased cellular expression of the natalizumab target *ITGA4* (CD49d) in the circulation of RRMS patients (Figure S3A). ITGA4 combines on cell surfaces with integrin beta 1 (*ITGB1*, CD29) to form the fully functional integrin very late antigen-4 (VLA-4) required for crossing of the BBB. Therefore, we confirmed that ITGB1 expression was also increased during treatment (Figures S3A and S3B). Importantly, *in vitro* exposure of immune cells to natalizumab did not affect ITGA4 and ITGB1 mRNA expression (Figures S3C and S3D),[7] suggesting the increase is not a direct drug effect but instead reflects an accumulation of CNS-homing immune cells in the peripheral blood. Next, we determined for each immune cell cluster and donor the extent to which the fraction of VLA-4+ CNS-homing cells increased during treatment with natalizumab (Figures 3A–3E). We observed the most significant increase and largest fold change in T cell cluster T09, which consisted largely of CD4+ effector memory T cells as well as a few central memory T cells (Figures 2C, 2E, 3E, and S2). The only other significant increase was observed in cluster T06, a close neighbor of T09 consisting mainly of CD4+ central memory T cells (Figures 2C, 2E, 3E, and S2); however, T06 showed a less pronounced increase of VLA-4+ cells. Thus, we selected cluster T09 for further exploration, as it consists of a mature effector CD4+ T cell population that seems to possess a strong drive to localize to the CNS in RRMS. To track CNS-homing cells in progressive MS, we sought to establish a way of measuring their presence in the blood independently of natalizumab. We conducted differential gene expression analysis for cluster T09 between natalizumab-treated and untreated time points (cohort MS1) to yield a CNS-homing signature (CNS-h) that marks these cells (Figures 4A and 4B). This signature included migration and adhesion factors, such as such as *ITGA4*, *AQP3*, *EZR*, *ADGRE5*, *RHOB*, and *RGS1*,[8–11] as well as encephalitogenic genes, such as *PIK3CG*, *KRAS*, *SOCS3*, and *TOB1* (Figures 4C and 4D; Table S1).[12–15] We first tested the enrichment of the CNS-h signature on expression data from the blood of the same RRMS patients (cohort MS1; untreated time points only) in comparison to matched healthy individuals (cohort HI1; Figure 5A). In this context, we observed a clear de-enrichment of the CNS-h signature in cluster T09, suggesting that we can measure a redistribution of CNS-homing cells from the periphery to the CNS in the peripheral blood. Notably, as the baseline frequencies of VLA4+ cells in T09 show interindividual variety at the same scale as the effect

(C) 5,000 randomly drawn cells per cohort are shown.
(D) 2,500 randomly drawn cells per group are shown.
(E) 500 randomly drawn cells per individual are shown. No color legend is provided because of the large number of individuals (n = 62).
(F) Messenger RNA (mRNA) expression of cell type marker genes projected on umap of all cohorts (MS1–3 and HI1–3).

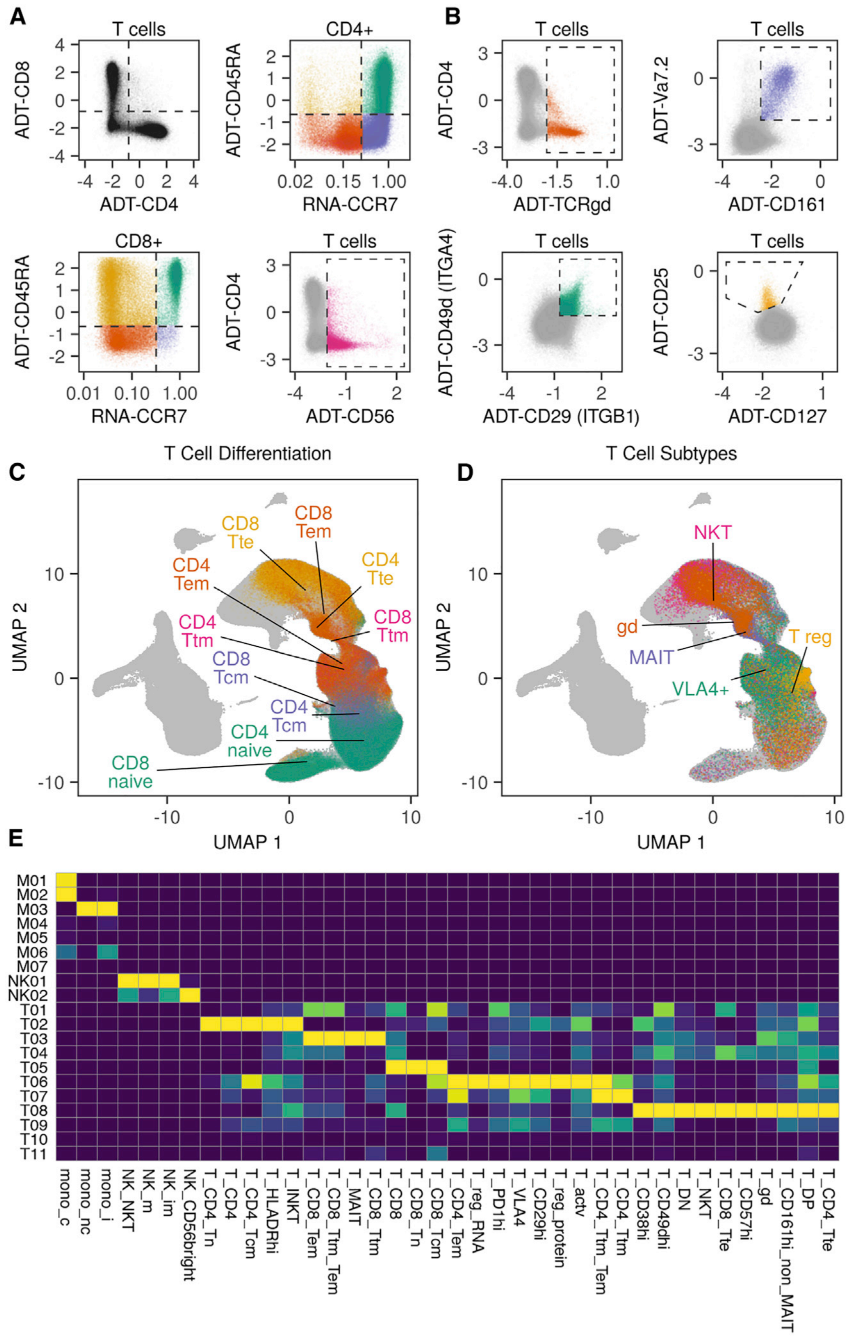

Figure 2. Integration of protein surface profiling for detailed mapping of canonical immune cell subtypes in the transcriptomic space

(A and B) Exemplary cell population gating based on sequenced surface marker profiles and imputed mRNA markers of n = 355,433 PBMCs (refer to Figure S1 for the complete gating strategy). Antibody-derived tags (ADTs) indicate that protein surface markers are shown, whereas "RNA" indicates imputed expression values based on the transcriptome.

(C and D) Projection of exemplary gated populations on umap (cohorts MS1–3 and HI 1–3). Refer to Figure S2 for projections of all gates. Lines point to the mean coordinates of the indicated populations.

(E) Column-scaled heatmap of gate distributions in unsupervised clusters. mono_c, classical monocytes (CD14+ monocytes); mono_i, intermediate monocytes; mono_nc, non-classical monocytes (CD16+ monocytes); NK_CD56bright, CD56-bright NK cells; NK_im, immature NK cells;

to be measured in this cross-sectional comparison, analysis based on cell frequencies would likely be inconclusive with the limited number of available samples (Figure 3E). Next, we validated our CNS-h signature on an independent cohort of 11 RRMS patients (cohort MS2) compared to 11 matched healthy individuals (cohort HI2). We confirmed a significant de-enrichment of the CNS-h signature in cluster T09 in the RRMS patients (Figure 5B). We then tested the CNS-h signature in a cohort of 10 progressive MS patients (cohort MS3) matched to 10 healthy individuals (cohort HI3). Again, we observed a significant de-enrichment of CNS-h in T09 cells (Figure 5C), suggesting that, not only in RRMS but also in progressive disease, these cells are still de-enriched in the peripheral immune compartment. In contrast, a randomly selected control gene signature of the same size as CNS-h was unchanged in all cohorts tested (Figures 5D–5F)

CNS-homing T09 cells localize to the white and gray matter in SPMS

Based on our observations in the peripheral blood of PPMS patients, we hypothesized that T cells originating from cluster T09 might be found in the brains of progressive MS patients. To test this, we used spatial RNA sequencing to examine the presence and localization of T09 cells in *post mortem* brain samples from SPMS patients. This technique enables enrichment testing of complete gene signatures in brain tissue with spatial resolution[16] and is therefore ideally suited to directly track T09 cells defined by a complex gene composition without loss of specificity (Figure 6A). We compiled a signature that distinguished T09 cells from all other immune cells in the blood based on our entire integrated dataset (cohorts MS1–3 and HI1–3; Table S1). We ensured that it clearly distinguished T09 cells in the blood of MS patients and that developmental brain-resident cells, such as microglia or astrocytes, did not enrich the T09 signature using a publicly available dataset with single-cell resolution (Figures 6B, S4A, and S4B).[17] Testing this devised T09 signature, we clearly observed a speckled enrichment pattern in brain tissue slices in four out of six MS patients, although no enrichment could be observed in brain tissue of four non-MS controls (Figures 6C–6E). Analyzing the spatial distribution of T09 enrichment, we noted an association with areas of white matter demyelination (Figure 6D). In addition, we observed a focal enrichment in the gray matter, often associated with sulcal areas or the border between gray and white matter (Figure 6E). Thus, we find direct evidence for an accumulation of T09 cells in the brains of MS patients, not only in white matter areas associated with relapses but also in gray matter areas strongly implicated in disease progression.[5]

T09 cells have an overlapping Th17-Tfh phenotype characterized by targetable surface markers

Having identified CNS-homing T09 cells in the brains of patients with progressive MS as well as in the blood of natalizumab-treated RRMS patients, we sought to

NK_m, mature NK cells; NK_NKT, NK T cells within NK cell clusters; T_actv, activated T cells; T_CD4, CD4+ T cells; T_CD4_Tcm, CD4+ central memory T cells; T_CD4_Tem, CD4+ effector memory T cells; T_CD4_Tn, naive CD4+ T cells; T_CD4_Tte, CD4+ terminal effector T cells; T_CD4_Ttm, CD4+ T transitional memory cells; T_CD4_Ttm_Tem, CD4+ T transitional memory/CD4+ T effector memory cells; T_CD8, CD8+ T cells; T_CD8_Tcm, CD8+ central memory T cells; T_CD8_Tem, CD8+ T effector memory cells; T_CD8_Tn, naive CD8+ T cells; T_CD8_Tte, CD8+ terminal effector T cells; T_CD8_Ttm, CD8+ T transitional memory cells; T_CD8_Ttm_Tem, CD8+ T transitional memory/CD8+ T effector memory cells; T_CD29hi, CD29-high T cells; T_CD38hi, CD38-high T cells; T_CD49dhi, CD49d-high T cells; T_CD57hi, CD57-high T cells; T_CD161hi_non_MAIT, CD161-high/non-MAIT cells; T_DN, CD4−/CD8− = double negative T cells; T_DP, CD4+/CD8+ = double positive T cells; T_gd, gamma-delta T cells; T_HLADRhi, HLADR-high T cells; T_INKT, invariant NK T cells; T_MAIT, mucosal-associated invariant T cells; T_NKT, NK T cells within T cell clusters; T_PD1hi, PD1-high T cells; T_reg_protein, T regulatory cells based on protein expression; T_reg_RNA, T regulatory cells based on RNA expression; T_VLA4, VLA4+ T cells.

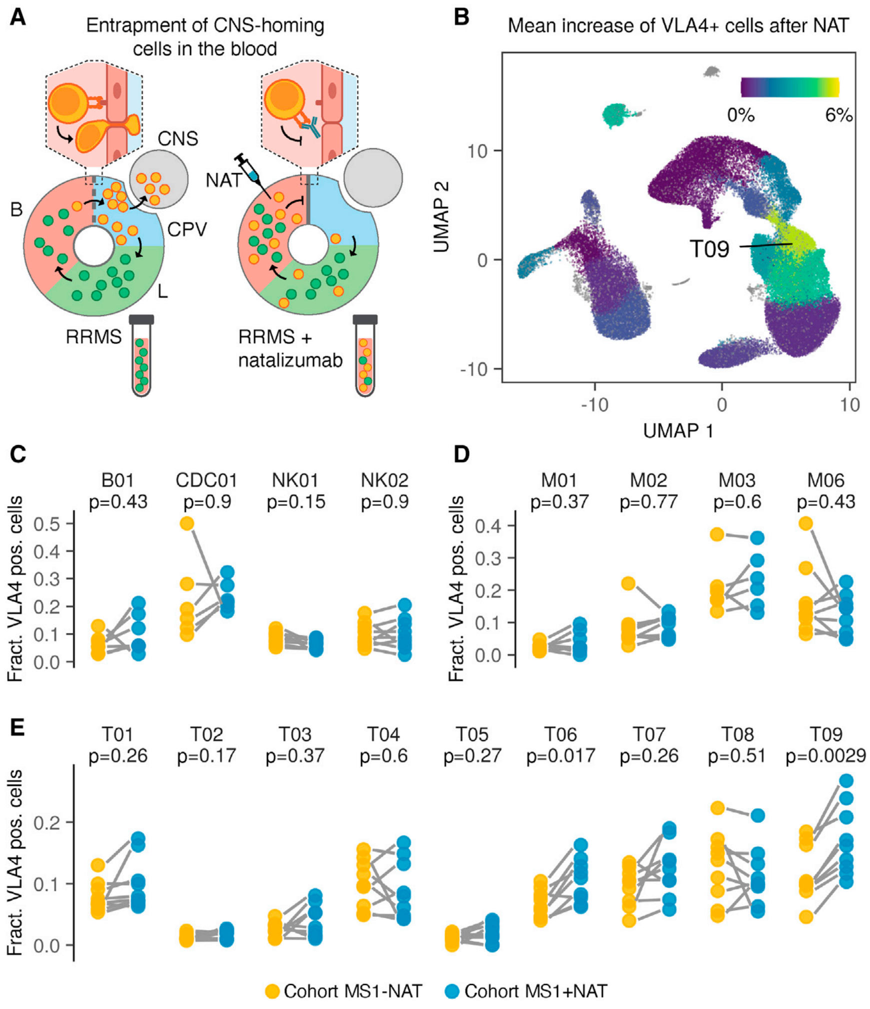

Figure 3. Entrapment of CNS-homing immune cells in the peripheral blood by natalizumab treatment uncovers their origin in cluster T09

(A) Experimental setup to identify CNS-homing immune cells in the blood (B). CNS-associated perivascular compartments (CPV), lymphatic system (L), and natalizumab treatment (NAT) are shown.

(B–E) Increase of VLA-4+ cells during natalizumab treatment compared to untreated time points (cohort MS1) in B cells (n = 7 RRMS patients), cDC (n = 6), monocytes (n = 9), NK cells (n = 9), and T cells (n = 9).

(B) Mean increase of VLA4+ cells during natalizumab treatment projected on umap.

(C) Relative increase of VLA4+ cells during natalizumab treatment for B cells, cDC, and NK cell subpopulations.

(D) Relative increase of VLA4+ cells during natalizumab treatment for monocyte subpopulations.

(E) Relative increase of VLA4+ cells during natalizumab treatment for T cell subpopulations.

FDR-adjusted paired two-tailed t tests were used in (C–E).

deeply characterize these cells to profile their potential as a therapeutic target. Based on their surface protein marker profile, T09 cells were mainly composed of CD4+ effector memory T cells (Figures 2C, 2E, and S2). To relate T09 cells to more specific T helper cell subtypes, we projected the transcriptomes of sorted CD4+ T cell populations[18] on our immune cell map (Figures 7A and S5A). Notably, we found that cluster T09, as a transcriptomic entity, transcended borders between classically defined T helper type 17 (Th17) and T follicular helper (Tfh) cells, suggesting that T09 cells have an overlapping phenotype between these cell types (Figure 7A). To better understand how this phenotype is defined, we derived marker

CellPress
OPEN ACCESS

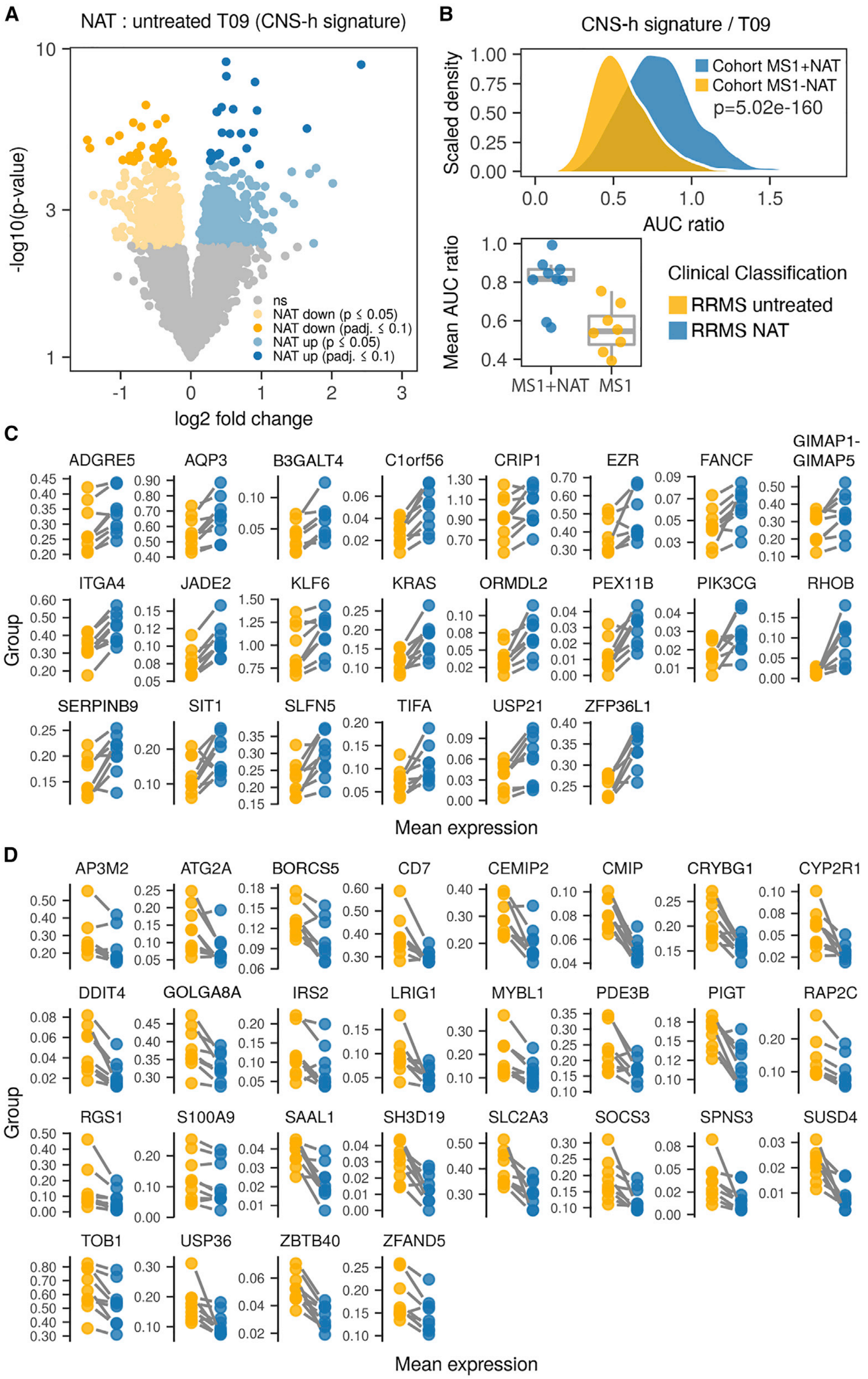

genes for T09 cells by contrasting them against other CD4+ memory cells and all other T cells (Figures 7B, 7C, and S5B–S5D; Table S1). T09 cells were most strongly distinguished from other CD4+ memory cells by their expression of killer cell lectin like receptor B1 (*KLRB1*; CD161) both on mRNA and protein level (Figures 7B, 7C, and S5B). We also observed the expected expression of CD161 in CD8+ and CD4/CD8-double-negative T cells (Tdn), including MAIT and Tgd (Figures 2D and S2), but T09 did not extend into these subsets (Figures 1B, 2D, and S2). In contrast to CD161, expression of lymphotoxin beta (*LTB*) was mostly restricted to CD4+ memory T cells, including T09, and was not seen in most CD8+ or Tdn cells (Figures 7C, 7D, and S6). Thereby, robust co-expression of *KLRB1* and *LTB* exclusively tagged T09 cells within the PBMC pool (Figure 7D). Additionally, we identified *AQP3*, *ICOS*, and *TNFRSF25* as markers with known surface expression suitable for discriminating T09 cells (Figures 7E and S6). MAF bZIP transcription factor (*MAF*) was the most prominently upregulated transcription factor in T09 (Figures 7B and 7E), which has been noted to play a dual role in Tfh and Th17 differentiation in mice,[19] followed by RAR-related orphan receptor A (*RORA*) (Figure 7E), which is the classical transcription factor defining Th17 cells.[20] A Gene Ontology (GO) enrichment analysis of T09 marker genes in comparison to other CD4+ memory T cells revealed upregulation of numerous closely related terms describing T cell migration, extravasation, chemotaxis, and cellular adhesion (Figure 7F; Table S2). Representative genes for the migration phenotype of T09 cells included *NINJ1*, *LIMS1*, *AQP3*, and *ITGA4* (Figures 7B, 7E, and S5C; Table S1).[21–23] In addition, T09 cells had a more differentiated phenotype than other CD4+ memory T cells, as indicated by downregulation of GO terms associated with cell cycle and T cell proliferation as well as upregulation of gene sets for terminally differentiated cells (Figure 7F; Table S2). Of note, in contrast to other CD4 memory cells, the top marker genes for cluster T09 included numerous genes, which have previously been proven to be encephalitogenic in MS animal models, such as *MAF*, *PDE4D*, *ABCB1*, *CCR1*, and *LGALS1*.[24–28] Together, cluster T09 consists of unique highly differentiated CD4+ T cells that possess traits of both Th17 and Tfh cells and express a rich repertoire of migratory and encephalitogenic factors, which make it plausible that they contribute to both the pathogenesis of RRMS and progressive MS (Figure 7G).

DISCUSSION

Here, we provide evidence that a population of CNS-homing CD4+ T cells (T09) that is relevant to the pathophysiology of RRMS is also detected in the CNS of patients with progressive disease. Intriguingly, we found that T09 cells were not only clustered in areas of white matter demyelination but were also present in the gray matter, including sulcal areas, where lymphocyte accumulations in progressive MS are mainly thought to occur.[5]

Figure 4. Differential gene expression analysis between T09 cells with and without natalizumab treatment identifies their CNS-homing signature
(A) Differentially expressed genes in cluster T09 between natalizumab-treated and untreated time points (cohort MS1), termed CNS-homing signature (CNS-h).
(B) Comparison of single-cell gene set enrichment of the CNS-h signature in cluster T09 for n = 150 cells per sample and mean gene set enrichment averaged for each sample (lower panel). n = 9 samples from natalizumab-treated RRMS patients and n = 8 samples from untreated time points of the same patients (cohort MS1) are shown.
(C and D) Mean mRNA expression in cluster T09 of indicated genes for n = 9 RRMS patients with and without natalizumab treatment (cohort MS1).
(C) Significantly upregulated genes with natalizumab treatment (adjusted p value of DEseq2 analysis $\leq$ 0.1).
(D) Significantly downregulated genes with natalizumab treatment (adjusted p value of DEseq2 analysis $\leq$ 0.1).
p values in (A, C, and D) are derived from differential gene expression analysis with DEseq2. False discovery rate (FDR)-adjusted paired two-tailed t tests were used in (B).

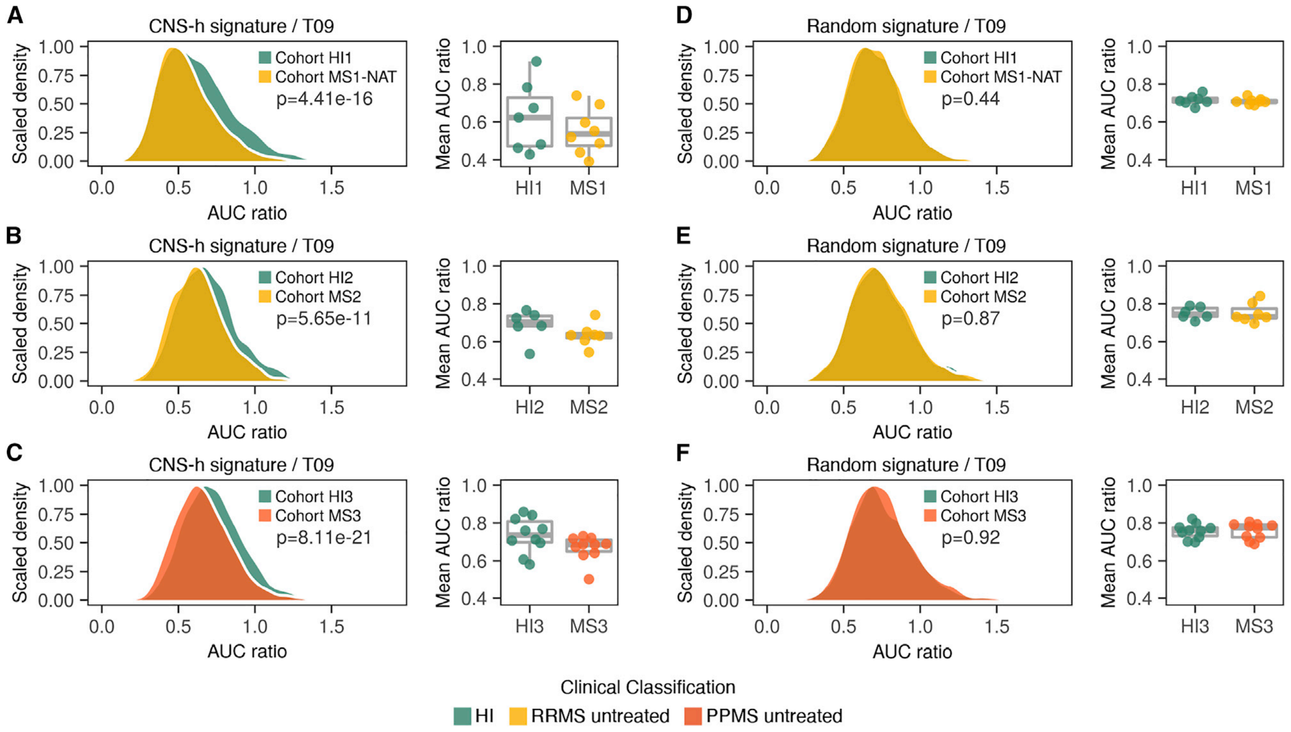

Figure 5. CNS-homing immune cells are reduced in the blood of relapsing-remitting and progressive MS patients

(A–F) Comparisons of single-cell gene set enrichment of the CNS-h signature (A–C) or a control signature of random genes (D–F) in cluster T09 for indicated cohorts with n = 150 cells per sample and mean gene set enrichment averaged for each sample (right panels).

(A and D) Samples from n = 8 RRMS patients without treatment (cohort MS1) compared with n = 7 HI (cohort HI1).

(B and E) Samples from n = 7 RRMS patients without treatment (cohort MS2) compared with n = 6 HI (cohort HI2).

(C and F) Samples from n = 10 PPMS patients without treatment (cohort MS3) compared with n = 10 HI (cohort HI3).

FDR-adjusted paired two-tailed t tests were used in (A)–(F).

Our data indicate that peripheral immune cells responsible for driving acute white matter demyelination during the RRMS phase become resident within cortical brain regions that are strongly associated with progression to disability. The presence of these cells, likely already at the initiation of disease but also later during the secondary progressive phase, raises the intriguing possibility that these cells play an active role in disease progression through colonization of the CNS. This observation may help to explain widespread clinical observations. Thus, it is known that immunomodulatory treatments, such as natalizumab, are effective during the relapsing-remitting phase of MS but that once secondary progressive disease has been established, they are unable to prevent the progression of disability.[2] Our data suggest that these treatments may not be effective during the progressive stage of disease because CNS-homing cells, although still detectable in the peripheral blood, will have already become resident within the CNS, where they may continue to drive inflammatory processes.

By taking a high-dimensional continuous immunophenotyping approach, we uncovered that these cells possess a distinct phenotype obscured in lower dimensional, discrete methods by bimodal attribution either as Th17 cells or Tfh cells. Thus, we defined a pathogenic cell population in RRMS and progressive MS that was difficult to fully capture in previous human studies. These cells are distinguished by expression of CD161 and LTB. CD161 has been described as a reliable marker of interleukin-17 (IL-17)-producing cells,[29] and variants have been

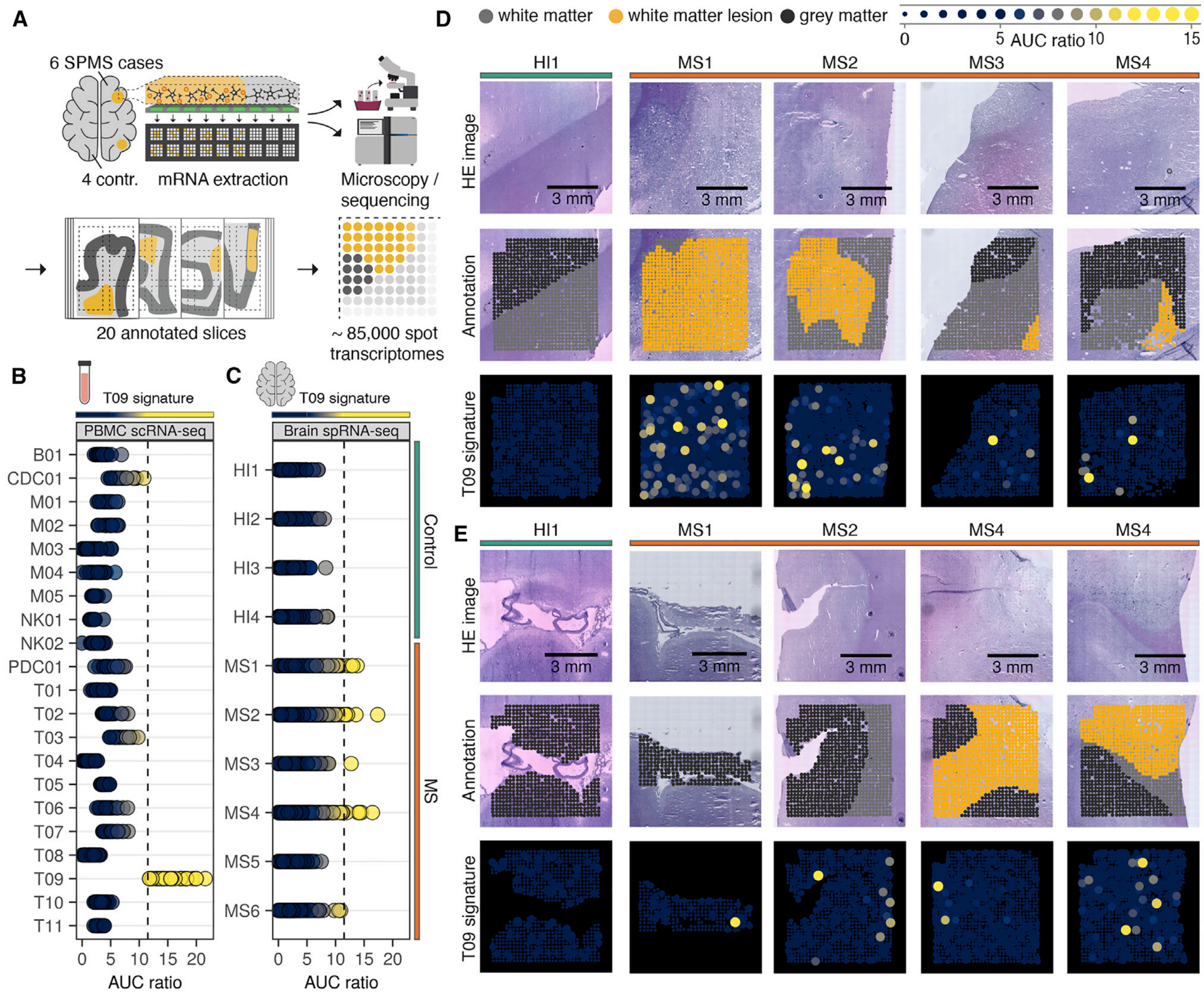

Figure 6. CNS-homing T09 cells localize to the white and gray matter in secondary progressive MS

(A) Schematic overview for spatial RNA sequencing of MS brain tissue.

(B) T09 signature enrichment for PBMC scRNA-seq data from n = 30 MS patients (cohorts MS1–3). Dots represent pseudobulks per sample. The dashed line indicates the cutoff chosen for signature enrichment (AUC ratio = 11.5), above which enrichment was found to be specific for cluster T09 within all PBMC populations.

(C) T09 signature enrichment for spatial RNA sequencing (spRNA-seq) data of brain slices from n = 4 control donors and n = 6 SPMS patients. Dots represent individual spot transcriptomes as indicated in (A).

(D and E) Representative spatial localization of T09 gene set enrichment in white matter (D) and gray matter (E).

associated with MS risk.[30] The combination of IL-17 production and LTB signaling has been associated with the induction of meningeal resident tertiary lymphatic tissue in experimental autoimmune encephalomyelitis (EAE) in mice,[31,32] implicating these pathways in the seeding of immune cell accumulations in MS. However, equivalent cells had not been identified in MS patients, and therefore, their therapeutic targeting was not feasible. By defining distinct migration factors and surface markers of LTB+/CD161+ T09 cells, a CNS-homing population that colonizes the CNS of progressive MS patients, we lay the groundwork for a therapeutic opportunity to target this pathomechanism. Additionally, a recent scRNA-seq study has provided further evidence for the importance of the Tfh phenotype in MS

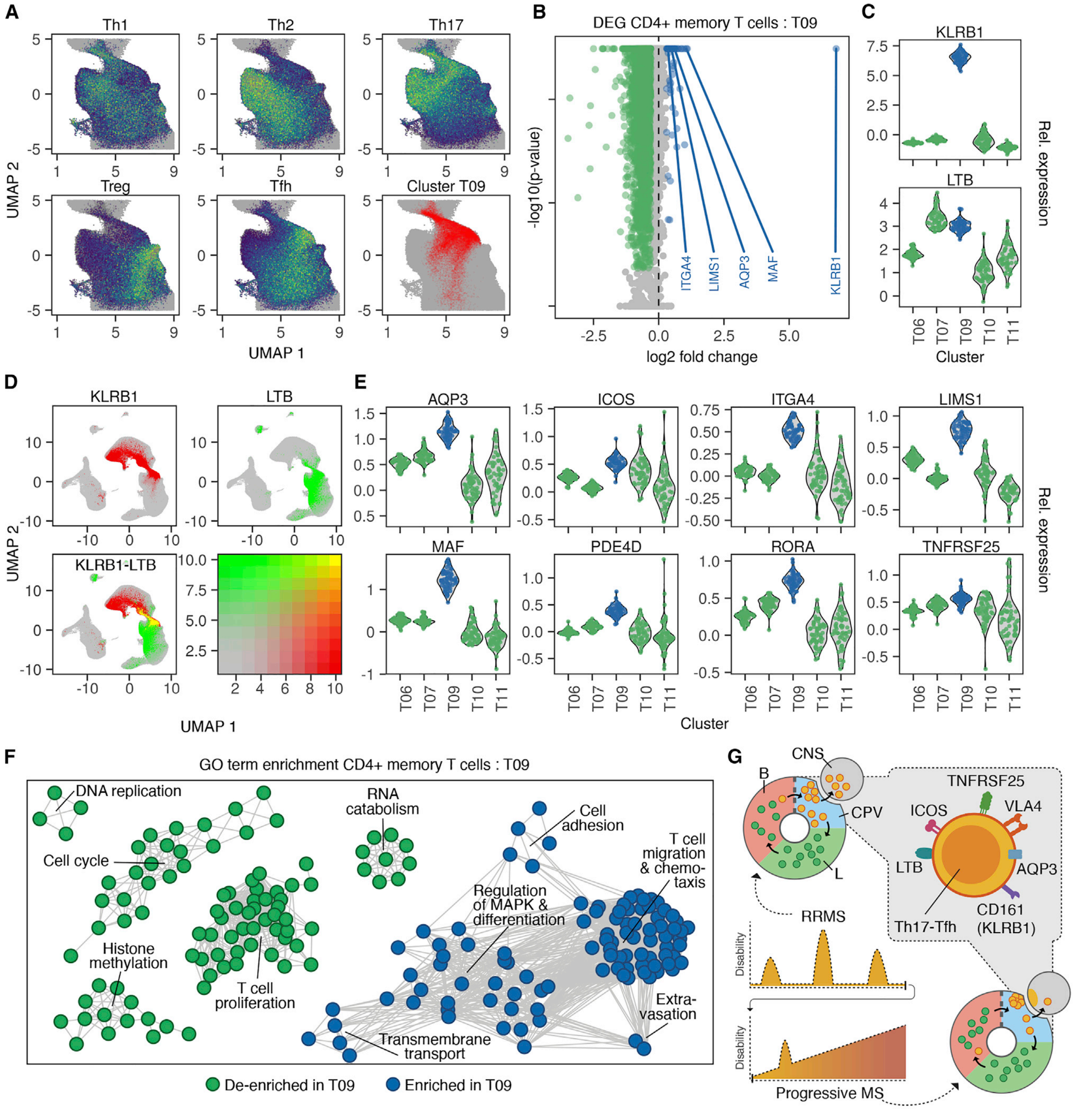

Figure 7. T09 cells have an overlapping Th17-Tfh phenotype characterized by targetable surface markers

(A) Gene set enrichment of T helper cell signatures derived from publicly available data[18] projected on umap plots of the CD4 memory T cell (CD4mem) compartment. The localization of T09 cells in CD4mem is indicated in the lower right panel. All cohorts (MS1–3 and HI1–3) are shown.

(B) Differential gene expression (DEG) analysis contrasting single-cell transcriptomes of T09 cells (n = 19,874) and other CD4mem cells (n = 73,002). All cohorts (MS1–3 and HI1–3) were used.

(C) Mean mRNA expression per individual (n = 62; cohorts MS1–3 and HI1–3) for KLRB1 and LTB in CD4mem clusters.

(D) (Co-)expression of KLRB1 and LTB projected on umap for all cohorts (MS1–3 and HI1–3).

(E) Mean mRNA expression per individual (n = 62; cohorts MS1–3 and HI1–3) for selected T09 markers in CD4mem clusters.

(F) Top 100 uniquely enriched or de-enriched GO terms in T09 markers from (B). Nodes indicate GO terms; edges represent shared genes between GO terms.

(G) Schematic indicating the surface marker profile of T09 cells homing to the CNS of RRMS and progressive MS patients.

with their demonstration of an enrichment of the Tfh signature in the cerebrospinal fluid of RRMS patients.[33]

Our findings have potentially important clinical implications, as they suggest that the initiation of an efficient immunomodulatory therapy in early MS could prevent peripheral immune cells from establishing residence within the CNS, which could in turn delay or halt the onset of the progressive stage of disease. Clinical support for this hypothesis has recently come from two retrospective studies suggesting that conversion to secondary progressive disease starts significantly earlier if the peripheral immune system is left unchecked.[34,35] However, long-term treatment with natalizumab and other high-efficacy immunomodulatory therapies is associated with serious side effects, such as potentially lethal JC virus reactivations and other severe infections[36,37]; this has led to the current paradigm of treatment escalation commonly applied to RRMS patients, which is considered low risk in terms of side effects to the patients yet fails to eventually curb disease progression. Thus, a therapeutic strategy to prevent CNS-homing immune cells from establishing themselves in the brain early in disease would have to be highly targeted to be practicable. Based on our study, it is attractive to speculate about a novel therapeutic approach for MS: utilizing short-term natalizumab "pre-conditioning" at the early stages of RRMS could trap the pathogenic T cell population in the easily accessible peripheral compartment, where it could then be specifically removed to potentially halt progressive disease onset. The identification of a number of surface protein targets on the CNS-homing T cell population in this study, including CD161, AQP3, LTB, ICOS, and TNFRSF25 (Figures 7C–7E, 7G, and S6), suggests possible options for developing tailored depletion therapies with tri-specific antibodies or combinatorial antigen-sensing cells, as has recently emerged for cancer therapy.[38,39] Together, we envisage that, by removing the CNS-homing T cells early in disease, it may be possible to prevent their establishment within the CNS and ultimately delay or prevent the transition to progressive disease.

Limitations of study

This work was aimed at studying the important process of immune cell colonization of the CNS in MS entirely in humans in order to maximize clinical relevance and translatability. Naturally, experiments in this setting cannot achieve the same degree of mechanistic depth as would be possible in an animal model. However, by investigating a defined molecular intervention to MS pathogenesis (antibody-mediated VLA-4 blockage) with detailed multimodal, multicohort immunophenotyping and direct analysis of the target tissue, we ultimately identify a promising avenue for preventing treatment-resistant progressive MS. Our study only represents the first step in developing such a new treatment. To make further progress, it will be important to better understand the temporal dynamics of CNS immune cell colonization and to assess the suitability of different surface proteins of T09 cells described in this study as targets for a depleting intervention.

Moreover, as we found that a comparison between untreated MS patients compared with healthy individuals did not show any significant differences in subpopulation frequencies (data not shown) and mostly identified low numbers of differentially expressed genes (Figures S7A–S7F; Table S1), many of which were driven by a small number of individuals, we cannot present a broader exploratory analysis of the MS immunophenotype. We believe this to be reflective of the high variability expected in cross-sectional human studies that are based on a relatively limited number of

tested individuals. Similar to the trend observed in gene-wide association studies, we imagine that a comprehensive and valid cell type resolved signature of the immune system in MS will only be derived from a much larger number of individuals. To this end, our dataset is a valuable starting point that can be incorporated into larger cohorts in the future.

STAR★METHODS

Detailed methods are provided in the online version of this paper and include the following:

- KEY RESOURCES TABLE
- RESOURCE AVAILABILITY
  - Lead contact
  - Materials availability
  - Data and code availability
- EXPERIMENTAL MODEL AND SUBJECT DETAILS
  - Study cohorts
- METHOD DETAILS
  - Single cell RNA sequencing and surface marker profiling
  - Data pre-processing for single cell analysis
  - Clustering and gating
  - Cell frequency analyses
  - Differential gene expression (DGE) analysis
  - Single cell gene set enrichment analysis
  - Immunohistochemistry and histology for neuropathological assessment of brain sections
  - Spatial RNA sequencing
  - T09 gene set enrichment analysis
  - Creation of T helper subtype marker gene sets
  - GO term enrichment
- QUANTIFICATION AND STATISTICAL ANALYSIS

SUPPLEMENTAL INFORMATION

Supplemental Information can be found online at https://doi.org/10.1016/j.medj.2021.01.006.

ACKNOWLEDGMENTS

We thank members of the Friese and Fugger laboratories for discussions and critical reading of the manuscript. We thank Sonja Loges and Nuray Akyüz for sequencing support and Marlon Stoeckius for technical advice in the establishment phase of CITE-seq. We thank Margaret Esiri and Dr. Gabriele De Luca for neuropathological evaluation of tissue slices. M.K. is supported by a Clinician-Scientist Fellowship from the Stifterverband für die Deutsche Wissenschaft. L.F. is supported by Wellcome (100308/Z/12/Z), the Medical Research Council UK (MC_UU_12010/3), the Oak Foundation (OCAY-15-520), and the NIHR Oxford BRC. M.A.F. is supported by intramural funds of the University Medical Center Hamburg-Eppendorf, the Deutsche Forschungsgemeinschaft (SFB1328, SPP1738, KFO296, FOR2289, FOR2879, and FOR5068), the Hamburg Ministry of Science and Research (LFF-FV-45), and the Bundesministerium für Bildung und Forschung-funded competence network of multiple sclerosis (KKNMS) and target validation.

AUTHOR CONTRIBUTIONS

M.K. compiled samples from the biobank of the Institute of Neuroimmunology and Multiple Sclerosis in Hamburg, Germany; designed, conjugated, and tested the

CITE-seq antibody panel; performed all data processing and computational analysis, including spatial RNA sequencing analysis; and prepared all figures of the article. H.E. established PBMC processing, scRNA-seq, CITE-seq, and cell hashing protocols and performed single-cell RNA sequencing experiments in this study. A.-L.S. performed spatial RNA sequencing experiments. J.B.E. helped with computational analysis. A.W. contributed to the design and validation of CITE-seq panels. N.K. performed auxiliary flow cytometry experiments and tests to assess cryopreservation effects on samples. C.S. contributed to data analysis. K.E.A. assisted with sample processing and sequencing. G.K. advised on scRNA-seq processing and experimental design. M.K., H.E., J.B.E., L.F., and M.A.F. designed the experiments for the study. M.K., H.E., J.B.E., L.F., and M.A.F. wrote the initial version of the manuscript, and A.-L.S. revised the manuscript. All co-authors contributed to the editing and discussion of the manuscript and approved the final version. L.F. and M.A.F. conceived, supervised, and funded the study.

DECLARATION OF INTERESTS

The authors declare no competing interests.

Received: November 11, 2020
Revised: December 17, 2020
Accepted: January 26, 2021
Published: February 16, 2021

REFERENCES

1. Dendrou, C.A., Fugger, L., and Friese, M.A. (2015). Immunopathology of multiple sclerosis. Nat. Rev. Immunol. *15*, 545–558.

2. Polman, C.H., O'Connor, P.W., Havrdova, E., Hutchinson, M., Kappos, L., Miller, D.H., Phillips, J.T., Lublin, F.D., Giovannoni, G., Wajgt, A., et al.; AFFIRM Investigators (2006). A randomized, placebo-controlled trial of natalizumab for relapsing multiple sclerosis. N. Engl. J. Med. *354*, 899–910.

3. Kapoor, R., Ho, P.-R., Campbell, N., Chang, I., Deykin, A., Forrestal, F., Lucas, N., Yu, B., Arnold, D.L., Freedman, M.S., et al.; ASCEND investigators (2018). Effect of natalizumab on disease progression in secondary progressive multiple sclerosis (ASCEND): a phase 3, randomised, double-blind, placebo-controlled trial with an open-label extension. Lancet Neurol. *17*, 405–415.

4. Scalfari, A., Romualdi, C., Nicholas, R.S., Mattoscio, M., Magliozzi, R., Morra, A., Monaco, S., Muraro, P.A., and Calabrese, M. (2018). The cortical damage, early relapses, and onset of the progressive phase in multiple sclerosis. Neurology *90*, e2107–e2118.

5. Magliozzi, R., Howell, O.W., Reeves, C., Roncaroli, F., Nicholas, R., Serafini, B., Aloisi, F., and Reynolds, R. (2010). A Gradient of neuronal loss and meningeal inflammation in multiple sclerosis. Ann. Neurol. *68*, 477–493.

6. Wegner, C., Esiri, M.M., Chance, S.A., Palace, J., and Matthews, P.M. (2006). Neocortical neuronal, synaptic, and glial loss in multiple sclerosis. Neurology *67*, 960–967.

7. Benkert, T.F., Dietz, L., Hartmann, E.M., Leich, E., Rosenwald, A., Serfling, E., Buttmann, M., and Berberich-Siebelt, F. (2012). Natalizumab exerts direct signaling capacity and supports a pro-inflammatory phenotype in some patients with multiple sclerosis. PLoS ONE *7*, e52208.

8. Martinelli, S., Chen, E.J.H., Clarke, F., Lyck, R., Affentranger, S., Burkhardt, J.K., and Niggli, V. (2013). Ezrin/Radixin/Moesin proteins and flotillins cooperate to promote uropod formation in T cells. Front. Immunol. *4*, 84.

9. Hamann, J., Veninga, H., de Groot, D.M., Visser, L., Hofstra, C.L., Tak, P.P., Laman, J.D., Boots, A.M., and van Eenennaam, H. (2010). CD97 in leukocyte trafficking. Adv. Exp. Med. Biol. *706*, 128–137.

10. Samuelsson, M., Potrzebowska, K., Lehtonen, J., Beech, J.P., Skorova, E., Uronen-Hansson, H., and Svensson, L. (2017). RhoB controls the Rab11-mediated recycling and surface reappearance of LFA-1 in migrating T lymphocytes. Sci. Signal. *10*, eaai8629.

11. Gibbons, D.L., Abeler-Dörner, L., Raine, T., Hwang, I.-Y., Jandke, A., Wencker, M., Deban, L., Rudd, C.E., Irving, P.M., Kehrl, J.H., and Hayday, A.C. (2011). Cutting edge: regulator of G protein signaling-1 selectively regulates gut T cell trafficking and colitic potential. J. Immunol. *187*, 2067–2071.

12. Comerford, I., Litchfield, W., Kara, E., and McColl, S.R. (2012). PI3Kγ drives priming and survival of autoreactive CD4(+) T cells during experimental autoimmune encephalomyelitis. PLoS ONE *7*, e45095.

13. Karussis, D., Abramsky, O., Grigoriadis, N., Chapman, J., Mizrachi-Koll, R., Niv, H., and Kloog, Y. (2001). The Ras-pathway inhibitor, S-trans-trans-farnesylthiosalicylic acid, suppresses experimental allergic encephalomyelitis. J. Neuroimmunol. *120*, 1–9.

14. Tanaka, K., Ichiyama, K., Hashimoto, M., Yoshida, H., Takimoto, T., Takaesu, G., Torisu, T., Hanada, T., Yasukawa, H., Fukuyama, S., et al. (2008). Loss of suppressor of cytokine signaling 1 in helper T cells leads to defective Th17 differentiation by enhancing antagonistic effects of IFN-gamma on STAT3 and Smads. J. Immunol. *180*, 3746–3756.

15. Schulze-Topphoff, U., Casazza, S., Varrin-Doyer, M., Pekarek, K., Sobel, R.A., Hauser, S.L., Oksenberg, J.R., Zamvil, S.S., and Baranzini, S.E. (2013). Tob1 plays a critical role in the activation of encephalitogenic T cells in CNS autoimmunity. J. Exp. Med. *210*, 1301–1309.

16. Ståhl, P.L., Salmén, F., Vickovic, S., Lundmark, A., Navarro, J.F., Magnusson, J., Giacomello, S., Asp, M., Westholm, J.O., Huss, M., et al. (2016). Visualization and analysis of gene expression in tissue sections by spatial transcriptomics. Science *353*, 78–82.

17. Schirmer, L., Velmeshev, D., Holmqvist, S., Kaufmann, M., Werneburg, S., Jung, D., Vistnes, S., Stockley, J.H., Young, A., Steindel, M., et al. (2019). Neuronal vulnerability and multilineage diversity in multiple sclerosis. Nature *573*, 75–82.

18. Monaco, G., Lee, B., Xu, W., Mustafah, S., Hwang, Y.Y., Carré, C., Burdin, N., Visan, L., Ceccarelli, M., Poidinger, M., et al. (2019). RNA-seq signatures normalized by mRNA abundance allow absolute deconvolution of human immune cell types. Cell Rep. *26*, 1627–1640.e7.

19. Bauquet, A.T., Jin, H., Paterson, A.M., Mitsdoerffer, M., Ho, I.-C., Sharpe, A.H., and Kuchroo, V.K. (2009). The costimulatory molecule ICOS regulates the expression of c-

Maf and IL-21 in the development of follicular T helper cells and TH-17 cells. Nat. Immunol. *10*, 167–175.

20. Yang, X.O., Pappu, B.P., Nurieva, R., Akimzhanov, A., Kang, H.S., Chung, Y., Ma, L., Shah, B., Panopoulos, A.D., Schluns, K.S., et al. (2008). T helper 17 lineage differentiation is programmed by orphan nuclear receptors ROR alpha and ROR gamma. Immunity *28*, 29–39.

21. Odoardi, F., Sie, C., Streyl, K., Ulaganathan, V.K., Schläger, C., Lodygin, D., Heckelsmiller, K., Nietfeld, W., Ellwart, J., Klinkert, W.E.F., et al. (2012). T cells become licensed in the lung to enter the central nervous system. Nature *488*, 675–679.

22. Cabodi, S., del Pilar Camacho-Leal, M., Di Stefano, P., and Defilippi, P. (2010). Integrin signalling adaptors: not only figurants in the cancer story. Nat. Rev. Cancer *10*, 858–870.

23. Hara-Chikuma, M., Chikuma, S., Sugiyama, Y., Kabashima, K., Verkman, A.S., Inoue, S., and Miyachi, Y. (2012). Chemokine-dependent T cell migration requires aquaporin-3-mediated hydrogen peroxide uptake. J. Exp. Med. *209*, 1743–1752.

24. Gabryšová, L., Alvarez-Martinez, M., Luisier, R., Cox, L.S., Sodenkamp, J., Hosking, C., Pérez-Mazliah, D., Whicher, C., Kannan, Y., Potempa, K., et al. (2018). c-Maf controls immune responses by regulating disease-specific gene networks and repressing IL-2 in $CD4^+$ T cells. Nat. Immunol. *19*, 497–507.

25. González-García, C., Bravo, B., Ballester, A., Gómez-Pérez, R., Eguiluz, C., Redondo, M., Martínez, A., Gil, C., and Ballester, S. (2013). Comparative assessment of PDE 4 and 7 inhibitors as therapeutic agents in experimental autoimmune encephalomyelitis. Br. J. Pharmacol. *170*, 602–613.

26. Kooij, G., Backer, R., Koning, J.J., Reijerkerk, A., van Horssen, J., van der Pol, S.M.A., Drexhage, J., Schinkel, A., Dijkstra, C.D., den Haan, J.M.M., et al. (2009). P-glycoprotein acts as an immunomodulator during neuroinflammation. PLoS ONE *4*, e8212.

27. Rottman, J.B., Slavin, A.J., Silva, R., Weiner, H.L., Gerard, C.G., and Hancock, W.W. (2000). Leukocyte recruitment during onset of experimental allergic encephalomyelitis is CCR1 dependent. Eur. J. Immunol. *30*, 2372–2377.

28. Wang, J., Lu, Z.-H., Gabius, H.-J., Rohowsky-Kochan, C., Ledeen, R.W., and Wu, G. (2009). Cross-linking of GM1 ganglioside by galectin-1 mediates regulatory T cell activity involving TRPC5 channel activation: possible role in suppressing experimental autoimmune encephalomyelitis. J. Immunol. *182*, 4036–4045.

29. Maggi, L., Santarlasci, V., Capone, M., Peired, A., Frosali, F., Crome, S.Q., Querci, V., Fambrini, M., Liotta, F., Levings, M.K., et al. (2010). CD161 is a marker of all human IL-17-producing T-cell subsets and is induced by RORC. Eur. J. Immunol. *40*, 2174–2181.

30. Søndergaard, H.B., Sellebjerg, F., Hillert, J., Olsson, T., Kockum, I., Lindén, M., Mero, I.-L., Myhr, K.-M., Celius, E.G., Harbo, H.F., et al. (2011). Alterations in KLRB1 gene expression and a Scandinavian multiple sclerosis association study of the KLRB1 SNP rs4763655. Eur. J. Hum. Genet. *19*, 1100–1103.

31. Pikor, N.B., Astarita, J.L., Summers-Deluca, L., Galicia, G., Qu, J., Ward, L.A., Armstrong, S., Dominguez, C.X., Malhotra, D., Heiden, B., et al. (2015). Integration of Th17- and lymphotoxin-derived signals initiates meningeal-resident stromal cell remodeling to propagate neuroinflammation. Immunity *43*, 1160–1173.

32. Peters, A., Pitcher, L.A., Sullivan, J.M., Mitsdoerffer, M., Acton, S.E., Franz, B., Wucherpfennig, K., Turley, S., Carroll, M.C., Sobel, R.A., et al. (2011). Th17 cells induce ectopic lymphoid follicles in central nervous system tissue inflammation. Immunity *35*, 986–996.

33. Schafflick, D., Xu, C.A., Hartlehnert, M., Cole, M., Schulte-Mecklenbeck, A., Lautwein, T., Wolbert, J., Heming, M., Meuth, S.G., Kuhlmann, T., et al. (2020). Integrated single cell analysis of blood and cerebrospinal fluid leukocytes in multiple sclerosis. Nat. Commun. *11*, 247.

34. Brown, J.W.L., Coles, A., Horakova, D., Havrdova, E., Izquierdo, G., Prat, A., Girard, M., Duquette, P., Trojano, M., Lugaresi, A., et al.; MSBase Study Group (2019). Association of initial disease-modifying therapy with later conversion to secondary progressive multiple sclerosis. JAMA *321*, 175–187.

35. He, A., Merkel, B., Brown, J.W.L., Zhovits Ryerson, L., Kister, I., Malpas, C.B., Sharmin, S., Horakova, D., Kubala Havrdova, E., Spelman, T., et al.; MSBase study group (2020). Timing of high-efficacy therapy for multiple sclerosis: a retrospective observational cohort study. Lancet Neurol. *19*, 307–316.

36. Major, E.O., Yousry, T.A., and Clifford, D.B. (2018). Pathogenesis of progressive multifocal leukoencephalopathy and risks associated with treatments for multiple sclerosis: a decade of lessons learned. Lancet Neurol. *17*, 467–480.

37. Luna, G., Alping, P., Burman, J., Fink, K., Fogdell-Hahn, A., Gunnarsson, M., Hillert, J., Langer-Gould, A., Lycke, J., Nilsson, P., et al. (2020). Infection risks among patients with multiple sclerosis treated with fingolimod, natalizumab, rituximab, and injectable therapies. JAMA Neurol. *77*, 184–191.

38. Wu, L., Seung, E., Xu, L., Rao, E., Lord, D.M., Wei, R.R., Cortez-Retamozo, V., Ospina, B., Posternak, V., Ulinski, G., et al. (2020). Trispecific antibodies enhance the therapeutic efficacy of tumor-directed T cells through T cell receptor co-stimulation. Nat. Can. *1*, 86–98.

39. Roybal, K.T., Rupp, L.J., Morsut, L., Walker, W.J., McNally, K.A., Park, J.S., and Lim, W.A. (2016). Precision tumor recognition by T cells with combinatorial antigen-sensing circuits. Cell *164*, 770–779.

40. Parekh, S., Ziegenhain, C., Vieth, B., Enard, W., and Hellmann, I. (2018). zUMIs - a fast and flexible pipeline to process RNA sequencing data with UMIs. Gigascience *7*, 1–9.

41. Li, H., Handsaker, B., Wysoker, A., Fennell, T., Ruan, J., Homer, N., Marth, G., Abecasis, G., and Durbin, R.; 1000 Genome Project Data Processing Subgroup (2009). The Sequence Alignment/Map format and SAMtools. Bioinformatics *25*, 2078–2079.

42. Dobin, A., Davis, C.A., Schlesinger, F., Drenkow, J., Zaleski, C., Jha, S., Batut, P., Chaisson, M., and Gingeras, T.R. (2013). STAR: ultrafast universal RNA-seq aligner. Bioinformatics *29*, 15–21.

43. Butler, A., Hoffman, P., Smibert, P., Papalexi, E., and Satija, R. (2018). Integrating single-cell transcriptomic data across different conditions, technologies, and species. Nat. Biotechnol. *36*, 411–420.

44. McGinnis, C.S., Murrow, L.M., and Gartner, Z.J. (2019). DoubletFinder: doublet detection in single-cell RNA sequencing data using artificial nearest neighbors. Cell Syst. *8*, 329–337.e4.

45. Hafemeister, C., and Satija, R. (2019). Normalization and variance stabilization of single-cell RNA-seq data using regularized negative binomial regression. Genome Biol. *20*, 296.

46. Wang, J., Agarwal, D., Huang, M., Hu, G., Zhou, Z., Ye, C., and Zhang, N.R. (2019). Data denoising with transfer learning in single-cell transcriptomics. Nat. Methods *16*, 875–878.

47. Love, M.I., Huber, W., and Anders, S. (2014). Moderated estimation of fold change and dispersion for RNA-seq data with DESeq2. Genome Biol. *15*, 550.

48. Wickham, H., Averick, M., Bryan, J., Chang, W., McGowan, L.D., François, R., Grolemund, G., Hayes, A., Henry, L., Hester, J., et al. (2019). Welcome to the Tidyverse. J. Open Source Softw. *4*, 1686.

49. Ewels, P.A., Peltzer, A., Fillinger, S., Patel, H., Alneberg, J., Wilm, A., Garcia, M.U., Di Tommaso, P., and Nahnsen, S. (2020). The nf-core framework for community-curated bioinformatics pipelines. Nat. Biotechnol. *38*, 276–278.

50. Aibar, S., González-Blas, C.B., Moerman, T., Huynh-Thu, V.A., Imrichova, H., Hulselmans, G., Rambow, F., Marine, J.-C., Geurts, P., Aerts, J., et al. (2017). SCENIC: single-cell regulatory network inference and clustering. Nat. Methods *14*, 1083–1086.

51. Yu, G., Wang, L.-G., Han, Y., and He, Q.-Y. (2012). clusterProfiler: an R package for comparing biological themes among gene clusters. OMICS *16*, 284–287.

52. Shannon, P., Markiel, A., Ozier, O., Baliga, N.S., Wang, J.T., Ramage, D., Amin, N., Schwikowski, B., and Ideker, T. (2003). Cytoscape: a software environment for integrated models of biomolecular interaction networks. Genome Res. *13*, 2498–2504.

53. Stoeckius, M., Hafemeister, C., Stephenson, W., Houck-Loomis, B., Chattopadhyay, P.K., Swerdlow, H., Satija, R., and Smibert, P. (2017). Simultaneous epitope and transcriptome measurement in single cells. Nat. Methods *14*, 865–868.

54. Stoeckius, M., Zheng, S., Houck-Loomis, B., Hao, S., Yeung, B.Z., Mauck, W.M., 3rd, Smibert, P., and Satija, R. (2018). Cell hashing with barcoded antibodies enables multiplexing and doublet detection for single cell genomics. Genome Biol. *19*, 224.

55. Crowell, H.L., Soneson, C., Germain, P.-L., Calini, D., Collin, L., Raposo, C., Malhotra, D., and Robinson, M.D. (2020). muscat detects subpopulation-specific state transitions from multi-sample multi-condition single-cell transcriptomics data. Nat. Commun. *11*, 6077.

56. Wong, K., Navarro, J.F., Bergenstråhle, L., Ståhl, P.L., and Lundeberg, J. (2018). ST Spot Detector: a web-based application for automatic spot and tissue detection for spatial Transcriptomics image datasets. Bioinformatics *34*, 1966–1968.

57. Jemt, A., Salmén, F., Lundmark, A., Mollbrink, A., Fernández Navarro, J., Ståhl, P.L., Yucel-Lindberg, T., and Lundeberg, J. (2016). An automated approach to prepare tissue-derived spatially barcoded RNA-sequencing libraries. Sci. Rep. *6*, 37137.

STAR★METHODS

KEY RESOURCES TABLE

REAGENT or RESOURCE	SOURCE	IDENTIFIER
Antibodies		
anti-CD4_clone-RPA-T4	Biolegend	CAT#300501; LOT#B226034; RRID: AB_314069
anti-CD8_clone-Hit8a	Biolegend	CAT#300901; LOT#B164480; RRID: AB_314105
anti-CD197 (CCR7)_clone-G043H7	Biolegend	CAT#353202; LOT#B199725; RRID: AB_10945157
anti-CD45RA_clone-HI100	Biolegend	CAT#304102; LOT#B198595; RRID: AB_314406
anti-CD45RO_clone-UCHL1	Biolegend	CAT#304202; LOT#B177325; RRID: AB_314418
anti-CD95_clone-DX2	Biolegend	CAT#305602; LOT#B206684; RRID: AB_314540
anti-CD27_clone-O323	Biolegend	CAT#302802; LOT#B186305; RRID: AB_314294
anti-CD28_clone-CD28.2	Biolegend	CAT#302901; LOT#B207053; RRID: AB_314303
anti-CD57_clone-HCD57	Biolegend	CAT#322302; LOT#B206682; RRID: AB_535988
anti-CD38_clone-HIT2	Biolegend	CAT#303502; LOT#B213310; RRID: AB_314354
anti-HLA-DR_clone-L243	Biolegend	CAT#307602; LOT#B217166; RRID: AB_314680
anti-CD49d (α4 integrin)_clone-9F10	Biolegend	CAT#304301; LOT#B185063; RRID: AB_314427
anti-CD29 (β1 integrin)_clone-TS2/16	Biolegend	CAT#303001; LOT#B200527; RRID: AB_314317
anti-CD194 (CCR4)_clone-1G1	BD	CAT#551121; LOT#6355945; RRID: AB_2074502
anti-CD25_clone-BC96	Biolegend	CAT#302602; LOT#B164472; RRID: AB_314272
anti-CD127_clone-A019D5	Biolegend	CAT#351302; LOT#B207381; RRID: AB_10718513
anti-CD183 (CXCR3)_clone-G025H7	Biolegend	CAT#353702; LOT#B218188; RRID: AB_10983073
anti-CD196 (CCR6)_clone-G034E3	Biolegend	CAT#353401; LOT#B148881; RRID: AB_10918626
anti-CCR10_clone-6588-5	Biolegend	CAT#341502; LOT#B199209; RRID: AB_1595419
anti-CXCR5_clone-J252D4	Biolegend	CAT#356901; LOT#B163804; RRID: AB_2561810
anti-TCR Vα24-Jα18_clone-6B11	Biolegend	CAT#342902; LOT#B204558; RRID: AB_2229301
anti-TCR γ/δ_clone-11F2	Nordic MUbio / Biozol	CAT#NMB-MUB1809P; LOT#7104; RRID: n/a
anti-TCR Vα7.2_clone-3C10	Biolegend	CAT#351702; LOT#B209777; RRID: AB_10900258
anti-CD161_clone-DX12	BD	CAT#556079; LOT#B7075709; RRID: AB_396346
anti-NKG2D_clone-1D11	Biolegend	CAT#320802; LOT#B228271; RRID: AB_492956
anti-CD159a (NKG2A)_clone-#131411	R&D	CAT#MAB1059-100; LOT#FVY0318061; RRID: AB_2280982
anti-CD19_clone-HIB19	Biolegend	CAT#302202; LOT#B250155; RRID: AB_314232
anti-IgD_clone-IA6-2	Biolegend	CAT#348202; LOT#B24390; RRID: AB_10550095

(Continued on next page)

Continued		
REAGENT or RESOURCE	SOURCE	IDENTIFIER
anti-CD24_clone-ML5	Biolegend	CAT#311102; LOT#B240399; RRID: AB_314851
anti-CD138_clone-DL-101	Biolegend	CAT#352302; LOT#B258491; RRID: AB_10915555
anti-CD14_clone-M5E2	Biolegend	CAT#301802; LOT#B257124; RRID: AB_314184
anti-CD16_clone-B73.1	Biolegend	CAT#360702; LOT#B243320; RRID: AB_2562693
anti-CD123/IL-3R_clone-6H6	Biolegend	CAT#306002; LOT#B254974; RRID: AB_314576
anti-CD11c_clone-3.9	Biolegend	CAT#301602; LOT#B252725; RRID: AB_314172
anti-CD56_clone-HCD56	Biolegend	CAT#318302; LOT#B254021; RRID: AB_604092
anti-CD279 (PD-1)_clone-EH12.2H7	Biolegend	CAT#329902; LOT#B250570; RRID: AB_940488
anti-CD152 (CTLA-4)_clone-BNI3	Biolegend	CAT#369602; LOT#B224872; RRID: AB_2566610
anti-CD3_clone-37895	Biolegend	CAT#317301; LOT#B201401; RRID: AB_571926
anti-CD298/β2-microglobulin-clone_LNH-94/2M2-hashtag_01	Biolegend	CAT#394601; LOT# B272595; RRID: AB_2750015
anti-CD298/β2-microglobulin-clone_LNH-94/2M2-hashtag_02	Biolegend	CAT#394603; LOT# B272602; RRID: AB_2750016
anti-CD298/β2-microglobulin-clone_LNH-94/2M2-hashtag_03	Biolegend	CAT#394605; LOT# B272598; RRID: AB_2750017
anti-CD298/β2-microglobulin-clone_LNH-94/2M2-hashtag_04	Biolegend	CAT#394607; LOT# B270523; RRID: AB_2750018
anti-CD298/β2-microglobulin-clone_LNH-94/2M2-hashtag_05	Biolegend	CAT#394609; LOT# B270803; RRID: AB_2750019
anti-CD298/β2-microglobulin-clone_LNH-94/2M2-hashtag_06	Biolegend	CAT#394611; LOT# B272604; RRID: AB_2750020
anti-CD298/β2-microglobulin-clone_LNH-94/2M2-hashtag_07	Biolegend	CAT#394613; LOT# B272606; RRID: AB_2750021
anti-CD298/β2-microglobulin-clone_LNH-94/2M2-hashtag_08	Biolegend	CAT#394615; LOT# B272605; RRID: AB_2750022
anti-CD298/β2-microglobulin-clone_LNH-94/2M2-hashtag_09	Biolegend	CAT#394617; LOT# B270545; RRID: AB_2750023
anti-CD298/β2-microglobulin-clone_LNH-94/2M2-hashtag_10	Biolegend	CAT#394619; LOT# B264719; RRID: AB_2750024
anti-CD298/β2-microglobulin-clone_LNH-94/2M2-hashtag_12	Biolegend	CAT#394623; LOT# B264718; RRID: AB_2750025
anti-CD298/β2-microglobulin-clone_LNH-94/2M2-hashtag_13	Biolegend	CAT#394625; LOT# B264717; RRID: AB_2750026
Biological samples		
Peripheral blood mononuclear cells of MS patients and healthy individuals	Biobank of the Institute of Neuroimmunology and Multiple Sclerosis (INIMS)	https://www.inims.de/#biobanking
Brain tissue of MS patients and control brain tissue	UK Multiple Sclerosis Society Tissue Bank	http://www.imperial.ac.uk/medicine/multiple-sclerosis-and-parkinsons-tissue-bank/
Critical commercial assays		
Chromium Single Cell Gene Expression 3′ v2	10X genomics	No longer commercially available
Chromium Single Cell Gene Expression 3′ v3	10X genomics	CAT#1000074, 1000092, 1000213
Library Preparation (LP) glass slides	Spatial Transcriptomics, Stockholm	The company is now part of 10X genomics. The original assay is no longer available (compare 10X Visium product)
Deposited data		
ScRNA-seq data of PBMCs from MS patients and healthy individuals	This paper	GEO: GSE144744
Bulk RNA-sequencing data of sorted PBMC populations	Ref18	GEO: GSE107011

(*Continued on next page*)

Continued

REAGENT or RESOURCE	SOURCE	IDENTIFIER
Bulk RNA-sequencing data of natalizumab-treated T cells	Ref7	GEO: GSE37783
Sc-RNA-seq data of brain tissue from MS patients and control tissue	Ref17	SRA: PRJNA544731
Oligonucleotides		
See Table S4		N/A
Software and algorithms		
bcl2fastq_v2.20.0.422	Illumina	https://support.illumina.com/sequencing/sequencing_software/bcl2fastq-conversion-software.html
zUMIs_v2.5.6b	[40]	https://github.com/sdparekh/zUMIs
samtools_v1.9	[41]	https://github.com/samtools/samtools
STAR_v2.6.0c	[42]	https://github.com/alexdobin/STAR
CITE-seq-Count_v1.4.2	https://doi.org/10.5281/zenodo.2590196	https://github.com/Hoohm/CITE-seq-Count
R_v3.6.1	The R Project for Statistical Computing	https://www.r-project.org/
Seurat_v3.1.1	[43]	https://satijalab.org/iocon/
DoubletFinder_v2.0.2	[44]	https://github.com/chris-mcginnis-ucsf/DoubletFinder
sctransform_v0.2.0	[45]	https://github.com/ChristophH/sctransform
SAVER-X_v1.0.0	[46]	https://github.com/jingshuw/SAVERX
Flowjo_v10.5.3	BD	https://www.flowjo.com/
DESeq2_v1.22.1	[47]	http://bioconductor.org/packages/release/bioc/html/DESeq2.html
Tidyverse_v1.2.1.9000	[48]	https://tidyverse.org
nf-core/rnaseq_v1.4.2	[49]	https://github.com/nf-core/rnaseq
AUCell_v1.4.1	[50]	https://bioconductor.org/packages/release/bioc/html/AUCell.html
clusterProfiler_v3.10.1	[51]	https://bioconductor.org/packages/release/bioc/html/clusterProfiler.html
Cytoscape_v3.6.1	[52]	https://cytoscape.org/
ST pipeline_v1.5.1	Spatial Transcriptomics	https://github.com/SpatialTranscriptomicsResearch/st_pipeline

RESOURCE AVAILABILITY

Lead contact

Further information and requests for resources and reagents should be directed to and will be fulfilled by the Lead Contact, Prof. Dr. Manuel A. Friese (manuel.friese@zmnh.uni-hamburg.de)

Materials availability

This study did not generate new unique reagents.

Data and code availability

All (unprocessed and processed) count matrices of scRNA-seq and CITE-seq experiments performed in this study are publicly available from Gene Expression Omnibus (GEO): GEO: GSE144744 including metadata and cell type annotations. Please note that the patient consent for this study did not cover the public deposition of raw sequencing data (fastq files) and raw data were therefore not uploaded to GEO. Access to raw sequencing data can be offered upon reasonable request following ethical approval at our institutions. Spatial RNA sequencing data and all code for data processing and analysis in R is available from the corresponding author on reasonable request. Other publicly available datasets used in this study are available from GEO (GEO: GSE107011, GEO: GSE37783) and from the Sequence Read Archive (SRA) (PRJNA544731).

EXPERIMENTAL MODEL AND SUBJECT DETAILS

Study cohorts

MS patients and healthy individuals were recruited through the MS outpatient clinic of the Department of Neurology, University Medical Center Hamburg-Eppendorf (UKE). The study was approved by the local ethics committee (Ethikkommission der Ärztekammer Hamburg, registration number PV4405) and informed consent was obtained from all patients and healthy individuals. All cohorts from which PBMCs were analyzed were compiled from cryopreserved samples collected in the biobank of the UKE Institute of Neuroimmunology and Multiple Sclerosis (INIMS). PBMCs were selected from n = 10 RRMS patients sampled during natalizumab treatment (cohort MS1). For n = 9 of these patients a second sample without natalizumab treatment was available and analyzed in the study. Additionally, PBMCs were used from n = 11 RRMS patients (cohort MS2) and n = 10 PPMS patients (cohort MS3) with active disease. These patients were not on any immunomodulatory medication at the time of sampling and only two PPMS patients from cohort MS3 had received any previous MS-specific long-term medication. PBMCs from n = 31 healthy individuals were carefully age- and sex-matched to the patients in this study (cohorts HI1–3). Further specifications of the cohorts are detailed in Table S3. For spatial RNA sequencing fresh-frozen brain tissue from MS patients or non-neurological controls were obtained from the UK Multiple Sclerosis Society Tissue Bank (registered charity 207495). This project falls under the umbrella of ethical approval obtained by the MS Society Tissue Bank (08/MRE09/31+5).

METHOD DETAILS

Single cell RNA sequencing and surface marker profiling

A panel of 38 antibodies (Key resources table; Table S4) was conjugated with barcoding oligonucleotides following the protocol for cellular indexing of transcriptomes and epitopes by sequencing (CITE-seq).[53] In addition, hashtag antibodies for sample multiplexing[54] were obtained from BioLegend (Key resources table; Table S4). PBMC samples were defrosted and assessed for viability, required to be > 95%. 200,000 cells per sample were blocked with Fc blocking reagent (FcX, BioLegend, cat. no. 422302) followed by staining with 0.5 μg of each prepared CITE-seq antibody and 0.5 μg of one hashtag antibody. Labeled single cell suspensions were mixed and 5,000–10,000 cells per sample were loaded onto a Chromium 10x controller (total load of 25,000–50,000 cells per well). All samples for a given donor and their matched control were loaded together and processed simultaneously. Samples were processed using the Chromium 3′ v3 (cohorts HI1 and MS1), v2 and v3 (cohorts HI2 and MS2), and v3 (cohorts HI3 and MS3) kits according to the manufacturer's instructions with modifications as described previously in the CITE-seq and cell hashing protocols.[53,54] Processed libraries were run on a Nextseq500 using an Illumina 75 cycle kit (R1 = 26, R2 = 55, i7 = 8, i5 = 0).

Data pre-processing for single cell analysis

Sequencing data was deconvoluted into separate libraries for cDNA, antibody derived tags (ADT) and cell hashing using bcl2fastq (Illumina). For cDNA libraries single cell barcode deconvolution and mapping was performed using the zUMIs pipeline[40] and the GRCh38 reference genome without alternative contigs. For ADT and cell hashing libraries barcode deconvolution was performed using CITE-seq-Count. Further data processing and analysis steps were carried out in R and handled within the Seurat single cell analysis framework.[43] First, cDNA and ADT samples were demultiplexed based on the signal of cell hashing antibodies using the *HTODemux* function from Seurat. In the process *inter*-sample doublets were identified and

removed. Next, single cell transcriptomes were subjected to the following basic quality filtering procedures: Genes expressed in less than ten cells were removed; pseudogenes and non-protein coding genes that were not annotated as either microRNAs or lincRNAs were discarded; highly abundant ribosomal protein coding genes were discarded as they are prone to carry batch effects and to be overrepresented in clustering and differential gene expression analysis; cells with less than 500 cDNA unique molecular identifiers (UMI) or more than 15,000 UMI counts were excluded as well as cells with less than 300 or more than 5,000 detected genes; furthermore, cells with less than 100 or more than 7,000 ADT UMI and cells with more than 20% mitochondrially encoded transcripts were excluded; remaining strong outliers on a plot of cDNA UMI count and number of detected genes were removed; automated *intra*-sample doublet detection was performed using DoubletFinder[44] and predicted doublets were removed. Next, each sample was normalized separately using sctransform,[45] followed by integration of all samples using Seurat as has been previously described[43] to correct for batch and biological group effects. A separate assay (termed SCT) was created in the Seurat object, in which all cells of the dataset were normalized together using sctransform and subjected to linear batch correction without correction for biological groups. This data was used in plots comparing gene expression between groups. Finally, pANN scores from DoubletFinder were visualized on umap plots of the integrated data and small clusters with overall high pANN considered as remaining *intra*-sample doublets were removed. The ADT data was reduced to cell barcodes for which single cell transcriptomes remained in the filtered transcriptomic dataset. Next, ADT normalization was performed across cells using centered log ratio transformations as described previously.[53] Selected markers were plotted for all samples separately to identify high quality stainings. 142 out of 204 ADT libraries showed excellent resolution, whereas the remaining profiles (all from one antibody conjugation batch) were considered to have inferior resolution. Tentative data integration including lower quality ADT profiles resulted in an overall reduced ability to gate subpopulations. Thus, they were not included in the final dataset. For 8 out of 38 surface markers low signal to noise ratio was observed in cryopreserved samples but not in fresh samples and these markers were not included in the final dataset. In analogy to the processing of the RNA data, ADT profiles were integrated and scaled using Seurat. To enable combined gating of protein surface marker data and RNA data we imputed dense gene expression matrices from the sparse raw data. Specifically, we performed machine-learning based imputation using SAVER-X[46] separately for each sample using a pretrained human immune cell model available from https://github.com/jingshuw/SAVERX (human_Immune.hdf5). The imputed gene expression data was integrated and scaled using Seurat. ADT and imputed RNA data for selected markers were exported as FCS files compatible with standard flow cytometry software.

Clustering and gating

Unsupervised transcriptomic clusters were identified with a shared nearest neighbor (SNN) modularity optimization-based algorithm implemented in Seurat using a resolution parameter of 0.75. Next, canonical RNA and ADT markers for the major cell types expected in PBMCs were visualized on a umap plot and clusters were annotated accordingly. Manual gating of cell subpopulations was performed for each major cell type on integrated ADT data and imputed RNA data within FlowJo. Cell IDs for each gate were exported from FlowJo into R to visualize the gated populations on umap plots.

Cell frequency analyses

The frequency of VLA4-postive cells was separately calculated for each sample as the number of ITGA4 and ITGB1 double positive cells in a given population (cell type or

subcluster). Combinations of samples and populations with less than 20 total available cells were excluded from the analysis together with their paired samples. Clusters with less than five samples remaining were excluded. The change in frequency under natalizumab treatment was calculated for each MS patient and cluster by subtracting the non-treatment frequencies from the frequencies in samples from natalizumab-treated time points. Statistical significance of frequency changes was assessed using FDR-adjusted paired two-tailed t tests.

Differential gene expression (DGE) analysis

Following recent suggestions for multi sample multi-condition scRNA-seq experiments[55], the DGE analysis for cluster T09 was performed in pseudo-bulks. First, separate expression matrices with raw UMI counts of cluster T09 were derived for each sample in cohort MS1. Before pseudo-bulking, each matrix was sampled (with replacement) to n = 1558 cells, the maximum number of cells found in a sample – cluster combination in our discovery cohort. This step was included, because the number of differentially expressed genes (DEG) was not independent of the number of input cells for pseudo-bulking (likely due to non-linear normalization steps of DEseq2). After sampling, the raw UMI counts in all matrices were aggregated gene-wise to pseudo-bulk samples. Next, DGE analysis was performed using DEseq2[47] with default settings. The design formula used was ~*donor_identity + group*, where group indicated the treatment status with natalizumab. Genes were considered to be differentially expressed at an FDR-adjusted p value $\leq$ 0.1. For gene sets used in gene set expression analysis also genes with a non-adjusted p value $\leq$ 0.05 were included. Cross-sectional DGE analyses (see Limitations of study, Figure S7; Table S1) were carried out in an identical fashion with the design formula adapted to ~*batch_pair + group*, where batch pair indicated the matched samples that were processed together and group distinguished between MS patients and healthy individuals. Markers for cluster T09 in contrast to all other T cells or in contrast to all other CD4+ memory T cells were determined based on the integrated transcriptomic data to describe this cell type independent of state changes in the different biological groups investigated. For this purpose, the *FindMarkers* function of Seurat was used to perform a Wilcoxon rank sum test. Genes were considered as markers for the cluster at an FDR-adjusted p value $\leq$ 0.1. For direct testing of differential mRNA expression of ITGA4 and ITGB1 (Figures S3A, S3C, and S3D) FDR-adjusted two-tailed t tests were conducted.

Single cell gene set enrichment analysis

Gene set enrichment analysis was performed using AUCell as previously described.[50] Signatures tested for enrichment were split in positive and negative marker gene sets. AUC values were then derived separately for positive and negative marker gene sets (Table S1). As an extension of the standard use of AUCell, the ratio of $AUC_{positive\ markers}$ / $AUC_{negative\ markers}$ was used to determine overall signature enrichment. AUC ratios for single cells were visualized as density plots. As a control for cross-sectional comparisons, random gene sets were composed, which contained equal numbers of genes as the positive and negative marker gene sets to be tested. Single cell AUC ratio values for the random control signature were derived as above.

Immunohistochemistry and histology for neuropathological assessment of brain sections

Cryosections were cut to a thickness of 10 μm and stained for expression of *MOG*, *HLA-DP/DQ/DR*, *GFAP*, and presence of oil red O (ORO) positivity according to standard protocols. Reagents used included anti-MOG supernatant (1:50 dilution)

(provided by Prof R. Reynolds, Imperial College London); DAKO rabbit polyclonal anti-GFAP (1:500 dilution) (Z0334, Agilent); DAKO mouse monoclonal anti-HLA-DP/DQ/DR (1:200 dilution) (CR3/43, Agilent); ImmPRESS HRP Anti-Mouse or Anti-Rabbit IgG (Peroxidase) Polymer Detection Kit (Vector Laboratories) and ImmPACT DAB Peroxidase (HRP) Substrate (Vector Laboratories); Oil Red O powder (Raymond A Lamb Ltd). The sections were neuropathologically evaluated by Prof Margaret Esiri (DM, FRCPath) and Dr Gabriele De Luca (MD, DPhil), University of Oxford.

Spatial RNA sequencing

The method was performed as described previously.[16] Briefly, cryosections of MS and control brain tissues were cut to a thickness of 10 μm and adhered to Library Preparation (LP) glass slides purchased from Spatial Transcriptomics, Stockholm, Sweden. This was followed by H&E staining and image acquisition, tissue permeabilization (1% Triton X followed by 0.1% pepsin (Sigma-Aldrich)) and overnight *in situ* cDNA synthesis. Tissues were then removed using Proteinase K solution (QIAGEN), followed by cDNA cleavage using a mix of Second Strand Buffer (1.1X) (Thermo Fisher Scientific), dNTPs (8.75 μM each) (Thermo Fisher Scientific), BSA (0.20 μg/μl) (NEB) and USER enzyme (0.1 U/μl) (NEB). The cleaved cDNA was collected and immediately stored at −20°C until further processing. Visualization of spatial spots for alignment with H&E images was achieved by hybridization with Cy3-labeled surface probes ([Cy3]AGATCGGAAGAGCGTCGTGT and [Cy3]GGTACAGAAGCGCGATAGCAG, IDT), followed by fluorescence image acquisition. Brightfield and fluorescent images were manually aligned using the ST Spot Detector tool described previously[56] on the basis of structural features detectable in both. Library preparation was carried out using a 2-phase robotic pipetting system (Magnatrix 8000+ Workstation, Magnetic Biosolutions AB) as described previously.[57] The robot was loaded with the following reagent mixtures. In phase 1 for second strand synthesis: First Strand Buffer (2.7X) (Thermo Fisher Scientific), DNA polymerase I (3.7 U/μl) (Thermo Fisher Scientific), RNase H (0.18 U/μl) (Thermo Fisher Scientific); for end blunting reaction: T4 DNA polymerase (3 U/μl) (NEB), EDTA (80 mM) (Thermo Fisher Scientific); for *in vitro* transcription: T7 NTP mix (7.5 mM each), T7 reaction buffer (1X), T7 enzyme mix (1X) (all part of Ambion MEGAscript T7 kit, Thermo Fisher Scientific), SUPERaseIn (1 U/μl) (Thermo Fisher Scientific). In phase 2 for adaptor ligation: aRNA ligation adaptor (0.71 μM) (IDT), T4 RNA Ligase Reaction Buffer (1X) (NEB), T4 RNA Ligase 2, truncated (20 U/μl) (NEB), RNase Inhibitor, murine (4 U/μl) (NEB), for second cDNA synthesis: reverse transcription primer (1.7 μM) (IDT), First Strand Buffer (2.5X) (Thermo Fisher Scientific), dNTP mix (0.83 mM each) (Thermo Fisher Scientific), DTT (12.5 mM) (Thermo Fisher Scientific), RNaseOut Recombinant Ribonuclease Inhibitor (5 U/μl) (Thermo Fisher Scientific), Superscript III (25 U/μl) (Thermo Fisher Scientific). Between all steps, libraries were purified using Agencourt RNAClean XP Beads (Beckman Coulter) as described previously.[57] Library indices were added with a PCR reaction. The number of cycles needed for indexing the libraries was determined by qPCR reaction in a total volume of 10 μL containing Kapa HiFi HotStart Ready Mix (1X) (Roche), EvaGreen (1X) (Biotium), PCR Primer InPE1.0 (0.5 μM) (IDT), PCR Primer InPE2.0 (0.01 μM) (IDT), 0.5 μM PCR Index (IDT) and 2 μL of purified cDNA. The following qPCR protocol was used: 98°C for 3 mins, followed by 25 cycles of 98°C for 20 s, 60°C for 30 s, 72°C for 30 s. Once the number of cycles needed for indexing each library was determined, libraries were indexed in a total reaction volume of 25 μL (5 μL cDNA + 20 μL reaction mix as above). The 6 libraries obtained from each ST slide were indexed with PCR indices 1-6 or 7-12. After indexing PCR, libraries were purified using Agencourt AMPure XP beads (Beckman Coulter) according to the manufacturer's protocol. The ratio of bead to sample volume used was 1.25:1. 80% ethanol was used for the wash steps.

The libraries were eluted in 20 µL RNase/DNase-free water. The average fragment length of the libraries was determined using a DNA High-Sensitivity kit (Agilent) and Bioanalyser 2100 according to the manufacturer's protocol. The cDNA concentration was measured by Qubit dsDNA HS assay (Thermo Fisher Scientific) according to the manufacturer's protocol. For sequencing, the 6 libraries from a slide were diluted to 4nM or 2nM (depending on the starting concentration of the least concentrated sample), pooled and prepared for paired-end sequencing (R1 30bp, R2 55bp) on the Illumina NextSeq 550 platform (Illumina) according to the manufacturer's protocol. Raw data processing was performed with the Spatial Transcriptomics pipeline including a mapping step with STAR and the GRCh38 reference genome.

T09 gene set enrichment analysis

Gene set enrichment analysis was performed as described above using AUCell with a marker signature that distinguished T09 cells in all PBMCs (Table S1). The analyses on PBMC data of MS patients from this study and brain cells from MS patients of a publicly available dataset[17] were carried out on pseudobulks of scRNA-seq data aggregated for each sample – cluster combination. Sample – cluster combinations with less than 20 cells and clusters with less than 3 available samples were excluded. Gene set enrichment analysis on spatial RNA sequencing data from MS post-mortem brain sections and control brain sections was carried out in the same fashion for each spot transcriptome. Resulting AUC ratios were compared with PBMC and brain residential cell clusters and their spatial distribution was plotted on tissue sections.

Creation of T helper subtype marker gene sets

A publicly available dataset,[18] which included bulk RNA sequencing data of sorted T helper cell subpopulations, was downloaded from GEO (GSE107011) as fastq files. Mapping and counting were performed using nf-core/rnaseq[49] with default parameters and GRCh38 as reference genome. DGE analysis contrasting different T helper cell subtypes was performed on the resulting count matrix with DeSeq2 to derive markers for each subtype. The resulting marker gene sets were validated on the sorted populations (Figure S5A).

GO term enrichment

As input for gene ontology (GO) enrichment analysis, marker genes for T09 in contrast to other CD4 memory T cells (see above) were selected, which had an FDR-adjusted p value ≤ 0.1 and an average logFC change > 0.3. GO term enrichment was tested using the *enrichGO* function from the clusterProfiler package.[51] GO terms with an FDR-adjusted p value ≤ 0.1 were considered to be significantly (de-)enriched. GO term enrichment maps were drawn for the top 100 uniquely enriched and de-enriched terms using the *emapplot* function from clusterProfiler. Go term maps were formatted in Cytoscape (https://cytoscape.org/) and manually annotated with group terms for clarity. Networks with less than four GO terms are not shown in Figure 7F. All significantly (de-)enriched GO terms are listed in Table S2.

QUANTIFICATION AND STATISTICAL ANALYSIS

To assess differences in single cell gene set enrichment (Figures 4B and 5A–5F) 150 cells were drawn from cluster T09 for each sample and group differences were tested using FDR-adjusted unpaired two-tailed t tests. Samples with less than 150 cells in cluster T09 were excluded from the comparison. All other statistical analyses are detailed in the respective sections of the article.

Med, Volume *2*

Supplemental information

Identifying CNS-colonizing T cells as potential therapeutic targets to prevent progression of multiple sclerosis

Max Kaufmann, Hayley Evans, Anna-Lena Schaupp, Jan Broder Engler, Gurman Kaur, Anne Willing, Nina Kursawe, Charlotte Schubert, Kathrine E. Attfield, Lars Fugger, and Manuel A. Friese

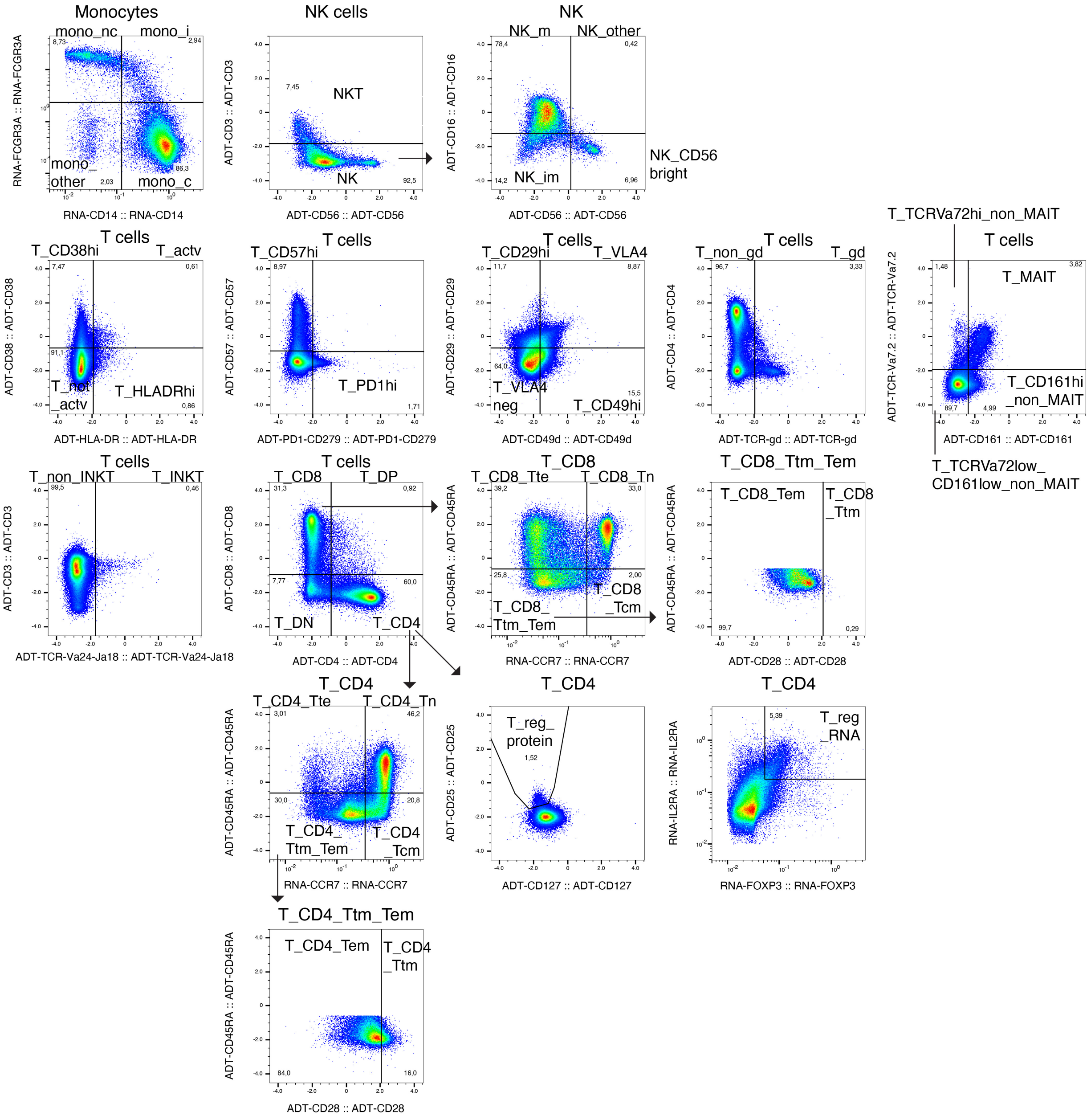

Figure S1. Gating strategy, related to Figure 2

Gating strategy based on sequenced surface marker profiles and imputed mRNA markers. Antibody-derived tags (ADT) indicate that protein surface markers are shown, whereas 'RNA' indicates imputed expression values based on the transcriptome. Abbreviations used: classical monocytes (mono_c, CD14+ monocytes), non-classical monocytes (mono_nc, CD16+ monocytes), intermediate monocytes (mono_i), NK T cells within NK cell clusters (NK_NKT), mature NK cells (NK_m), immature NK cells (NK_im), CD56-bright NK cells (NK_CD56bright), naïve CD4+ T cells (T_CD4_Tn), CD4+ T cells (T_CD4), CD4+ central memory T cells (T_CD4_Tcm), HLADR-high T cells (T_HLADRhi), invariant NK T cells (T_INKT), CD8+ T effector memory cells (T_CD8_Tem), CD8+ T transitional memory / CD8+ T effector memory cells (T_CD8_Ttm_Tem), mucosal-associated invariant T cells (T_MAIT), CD8+ T transitional memory cells (T_CD8_Ttm), CD8+ T cells (T_CD8), naïve CD8+ T cells (T_CD8_Tn), CD8+ central memory T cells (T_CD8_Tcm), CD4+ effector memory T cells (T_CD4_Tem), T regulatory cells based on RNA expression (T_reg_RNA), PD1-high T cells (T_PD1hi), VLA4+ T cells (T_VLA4), CD29-high T cells (T_CD29hi), T regulatory cells based on protein expression (T_reg_protein), activated T cells (T_actv), CD4+ T transitional memory / CD4+ T effector memory cells (T_CD4_Ttm_Tem), CD4+ T transitional memory cells (T_CD4_Ttm), CD38 -high T cells (T_CD38hi), CD49d-high T cells (T_CD49dhi), CD4–/CD8– = double negative T cells (T_DN), NK T cells within T cell clusters (T_NKT), CD8+ terminal effector T cells (T_CD8_Tte), CD57-high T cells (T_CD57hi), gamma-delta T cells (T_gd), CD161-high / non-MAIT cells (T_CD161hi_non_MAIT), CD4+/CD8+ = double positive T cells (T_DP), CD4+ terminal effector T cells (T_CD4_Tte).

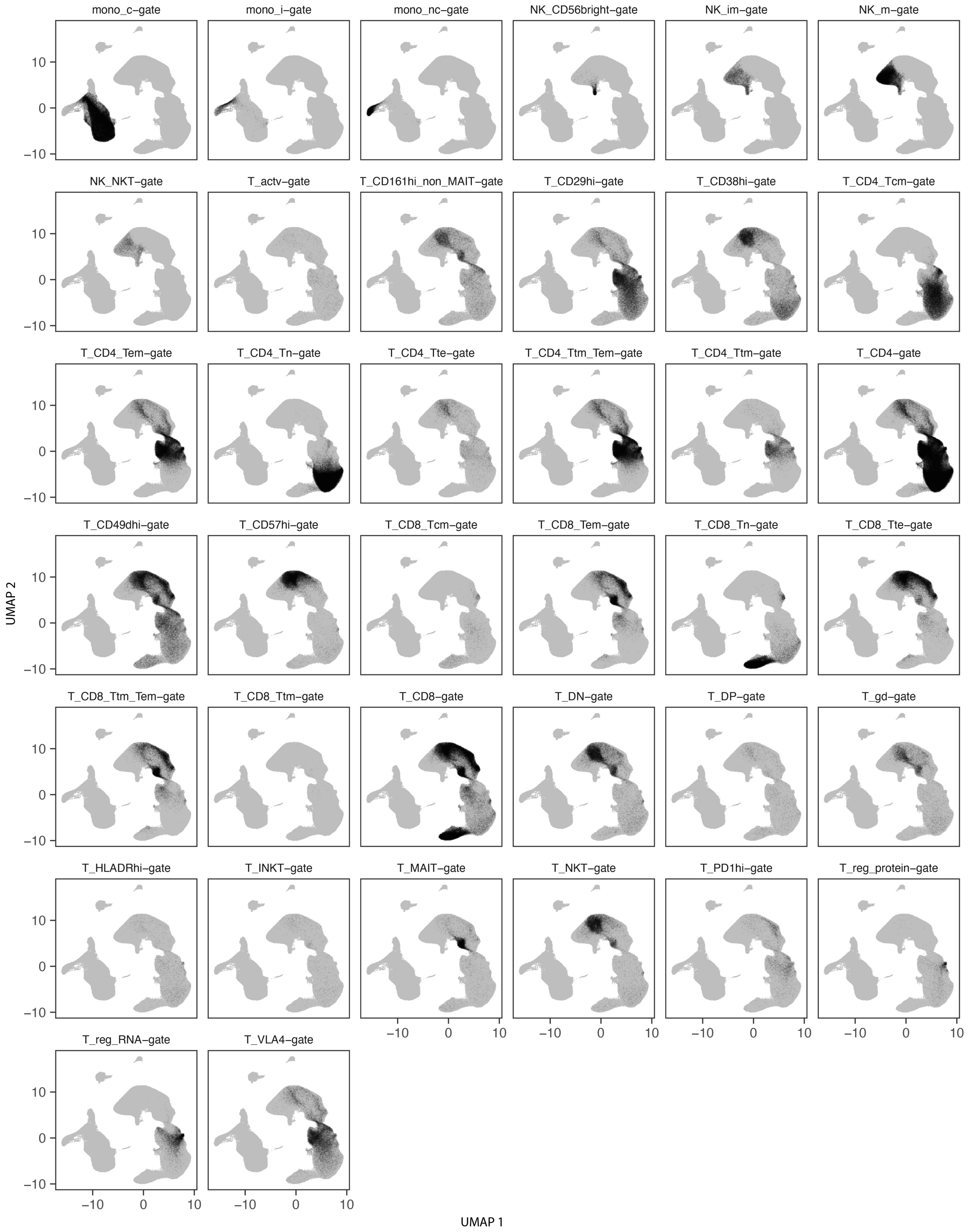

Figure S2. Projection of gates on immune cell map, related to Figure 2

Each gate shown in Figure S1 was projected on umap plots of all cohorts (MS1–3, HI1–3).

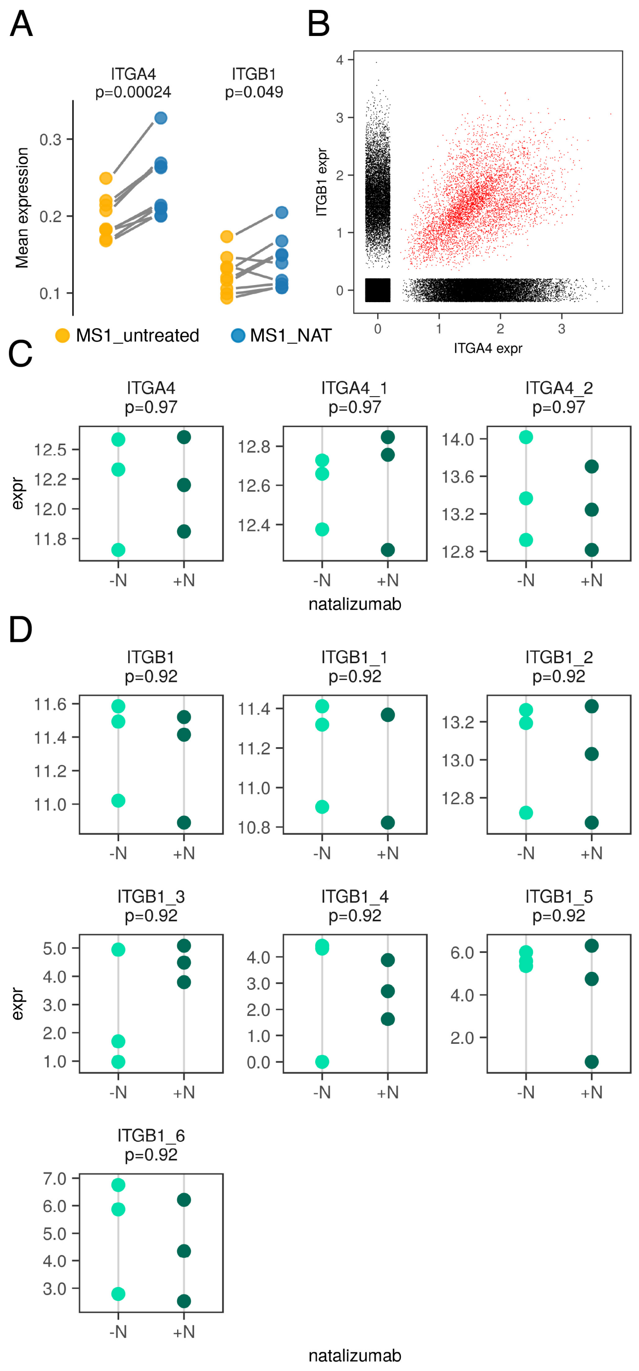

Figure S3. Integrin expression and VLA4 gating, related to Figure 3

(A) Mean expression of ITGA4 and ITGB1 in all PBMCs of *n* = 9 RRMS patients with and without natalizumab (NAT) treatment (cohort MS1).

(B) Expression of indicated integrins in single PBMCs (cohorts MS1–3, HI1–3). Jitter is added to the data to decrease overplotting. Cells gated as VLA4 positive are marked in red.

(C,D) Microarray-based mRNA expression of indicated integrins from publicly available data[7] in CD4+ T cells from *n* = 3 healthy donors cultured *in vitro* for 10 days with (+N) or without (–N) natalizumab.

FDR-adjusted paired two-tailed *t*-tests were used in (A). FDR-adjusted unpaired two-tailed *t*-tests were used in (C,D) as data pairs were not indicated in the available metadata.

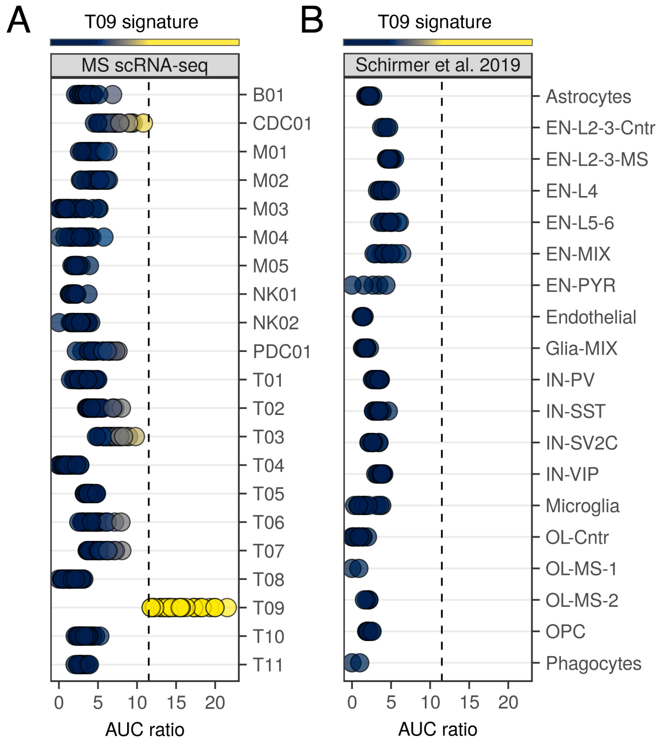

Figure S4. The T09 signature is not enriched in brain cells, related to Figure 6

(A–B) T09 signature enrichment for PBMC scRNA-seq data (A) from *n = 30* MS patients (cohorts MS1–3) and for brain single nuclei RNA sequencing data (B) of *n = 12* MS patients from a publicly available dataset[17].

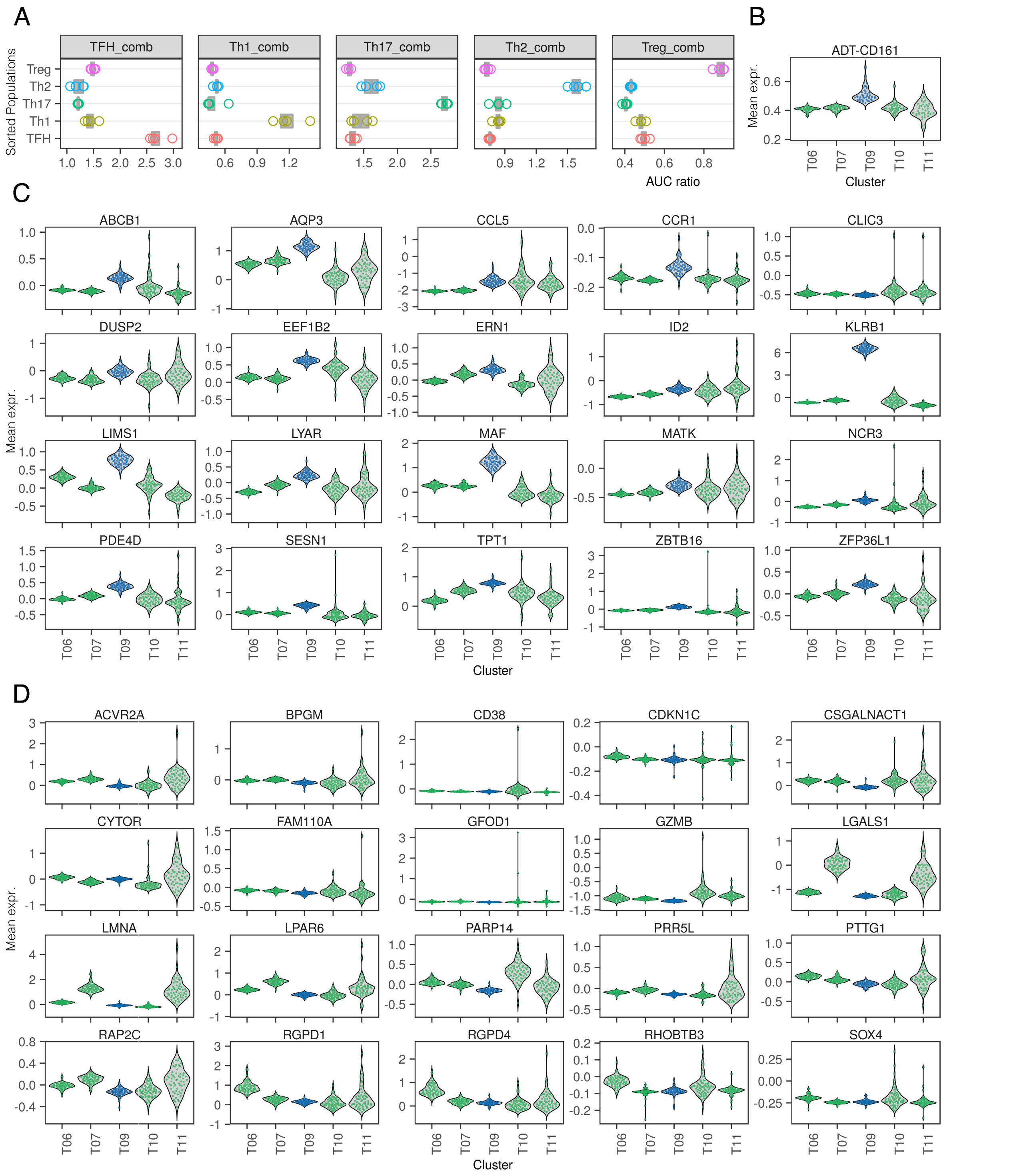

Figure S5. Gene set enrichment-based T helper cell subset discrimination and expression of T09 markers in CD4$^+$ memory T cell clusters, related to Figure 7

(A) Marker gene sets for T helper cell subsets were derived from publicly available data[18]. Gene set enrichment of each signature is shown on transcriptomes of sorted T helper cell populations from the same study. Box plots represent median and interquartile range (IQR).

(B) Mean protein surface expression of CD161 per individual (n = 42).

(C,D) Mean mRNA expression of indicated genes in CD4mem clusters for n = 62 individuals (cohorts MS1–3, HI1–3). (C) Top 20 positive markers for T09, (D) Top 20 negative markers for T09.

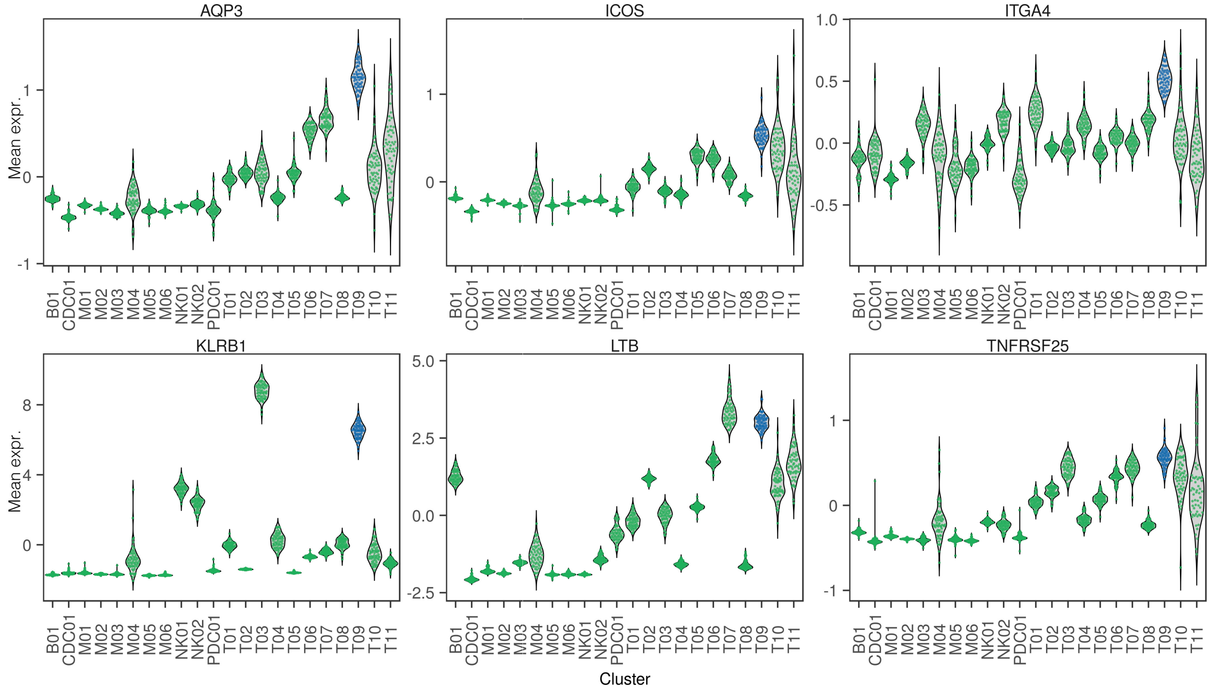

Figure S6. Expression of selected T09 surface markers in all PBMC clusters, related to Figure 7

Mean mRNA expression of indicated genes in indicated clusters for *n* = 62 individuals (cohorts MS1–3, HI1–3).

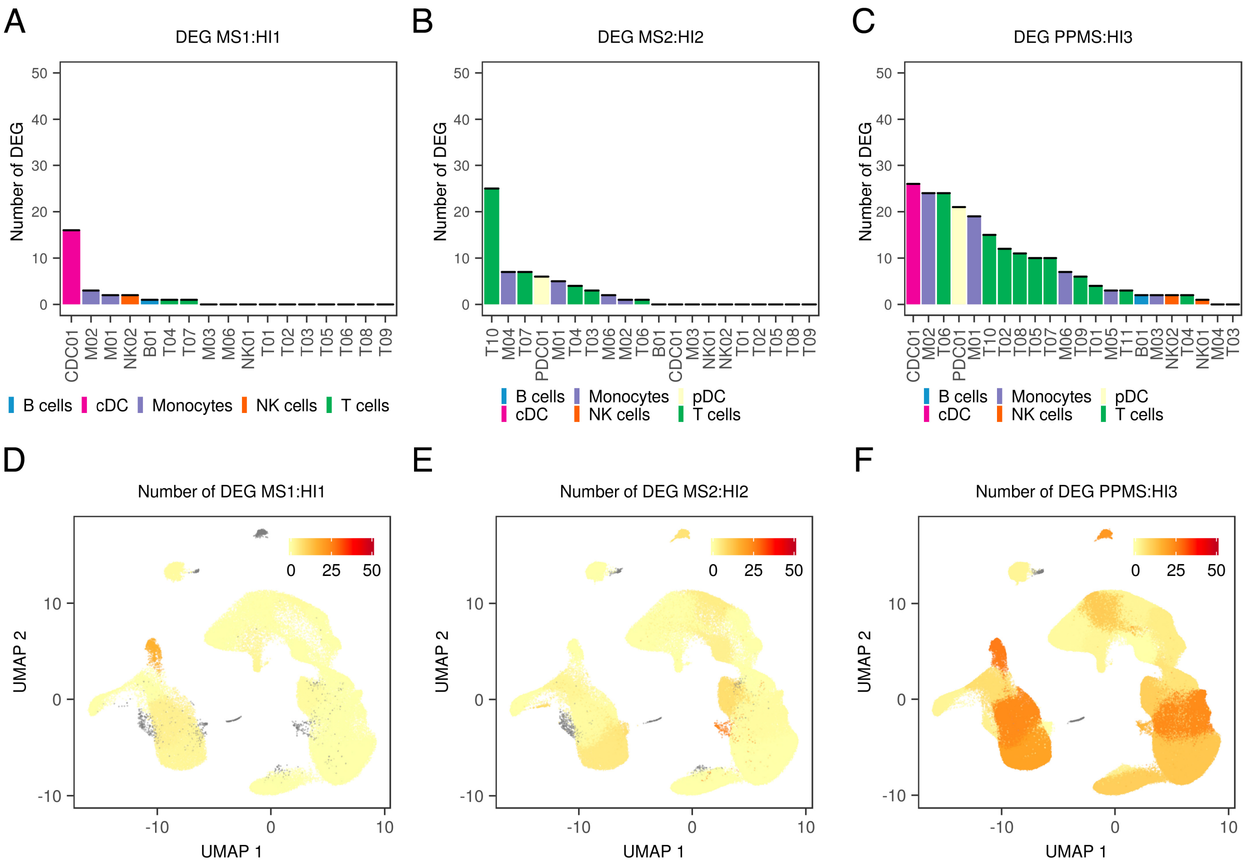

Figure S7. Cross-sectional differential gene expression analysis, related to Figure 4 and limitations of study

(A–C) Number of significantly (adjusted *P* values ≤ 0.1) differentially expressed genes (DEG) between MS patients and healthy individuals. (A) Comparison between cohorts MS1 and HI1, *n* = 19 for T01–04,06–09, NK01,02, M01,02,06, *n* = 18 for T05, M03, *n* = 16 for B01, *n* = 15 for CDC01. (B) Comparison between cohorts MS2 and HI2, *n* = 22 for T01–T09, NK01, M02, *n* = 21 for NK02, *n* = 20 for M01,03,06, *n* = 18 for B01, *n* = 17 for CDC01, *n* = 16 for PDC01, *n* = 15 for M04, *n* = 12 for T10. (C) Comparison between cohorts MS3 and HI3, *n* = 20 for T01–T10, NK01,02, M01–04,06, B01, CDC01, *n* = 19 for M05, PDC01, *n* = 16 for T11.

(D–F) Number of DEG in indicated comparisons projected on umap.
